# Study on the behavioral decision of multiple subjects of agricultural green production under the double carbon target in China

**DOI:** 10.3389/fpubh.2025.1575121

**Published:** 2025-07-30

**Authors:** Xiaoli Zhang, Xiuli Duan, Xinhe Zhou, Zhengfei Pang, Hongcheng Duan, Yun Teng, Hui Xu

**Affiliations:** ^1^School of Accounting, Harbin Finance University, Harbin, China; ^2^Research Center of Digital Economy and New Quality Productivity, Harbin Finance University, Harbin, China; ^3^School of Information Engineering, Heilongjiang Forestry Vocational-Technical College, Mudanjiang, China; ^4^College of Engineering, Northeast Agricultural University, Harbin, China; ^5^School of Management, Trinity Western University, Langley, BC, Canada

**Keywords:** dual carbon targets, agricultural carbon peaking and carbon neutrality, agricultural green production, behavioral decision making, evolutionary game

## Abstract

Excessive carbon emissions constitute a major driver of contemporary global warming. Achieving carbon neutrality in agriculture, particularly via carbon peaking, represents a critical strategy for emission reduction, wherein green agricultural production serves as a pivotal component. This study constructs a unified model encompassing the government, agricultural enterprises, and farms engaged in green agricultural production, utilizing a dynamic evolutionary game approach to examine the decision-making behaviors of these stakeholders. The findings indicate that green agricultural production entails the responsibilities of managers, users, and producers. The government fulfills a guiding and supervisory role, while agricultural enterprises actively produce low-carbon agricultural materials, and farms rigorously implement these materials. To facilitate this, the government should implement a reward and punishment mechanism, including increased carbon tax rebates for enterprises producing low-carbon materials and subsidies for farms utilizing them. Conversely, penalties should be levied on entities that fail to comply with low-carbon practices. The government must meticulously calibrate subsidies and fines within a reasonable range, appropriately reduce taxes, and effectively manage regulatory costs to mitigate financial strain. Under government incentives and penalties, agricultural enterprises should proactively respond by offering price concessions to farms utilizing low-carbon materials, balancing costs and benefits, and fostering a socially responsible corporate image. Farms should establish close collaboration with the government and enterprises to ensure the procurement, utilization, and production of low-carbon agricultural materials. This study provides valuable insights for advancing agricultural carbon neutrality through the perspective of green agricultural production.

## 1 Introduction

Achieving carbon peaking and carbon neutrality represents a critical global imperative (1). The accelerating pace of industrialization and technological innovation has led to substantial greenhouse gas (GHG) emissions, contributing to a consistent rise in global temperatures. Global average temperatures have reached record highs, exceeding pre-industrial levels by 1.2°, with the period from 2016 to 2019 registering as the three warmest years on record (2). Excessive carbon emissions are a primary driver of this crisis, making the reduction of these emissions an essential strategy for addressing environmental challenges and achieving carbon neutrality. In this context, “carbon” refers to carbon dioxide and other greenhouse gases, while “neutrality” signifies a balance between emissions and absorption, resulting in net-zero emissions (3). Climate change poses a shared challenge for humanity and impacts the well being of future generations. Consequently, carbon neutrality has emerged as a crucial initiative recognized globally as a means to protect the environment (4). At the Leaders' Climate Summit on April 22–23, 2021, developed regions, including the United States, Europe, and Japan, established ambitious carbon neutrality and emission reduction targets. President Xi Jinping reaffirmed China's commitment to achieving the strategic goals of “peaking carbon emissions by 2030 and achieving carbon neutrality by 2060” (5). A 2022 report by the United Nations Environment Programme indicated that over 120 countries had made carbon-neutral commitments (6). Globally, nations are prioritizing their dual carbon targets at a strategic level (7).

Agricultural carbon emissions constitute a significant proportion of global carbon emissions (8). As the second-largest source globally (9, 10), agriculture accounts for ~10%−12% of total emissions (11, 12). China, a major agricultural nation, has experienced a substantial increase in greenhouse gas (GHG) emissions from its agricultural activities (13). According to China's GHG emissions data, total national emissions in 2014 were ~11.2 billion tons, with agricultural emissions contributing 830 million tons, or 7.4% of the total (14, 15). Within non-CO_2_ GHG emissions, agriculture accounted for 48%, comprising 22.6% methane from paddy fields, 34.7% nitrous oxide from nitrogen fertilizers, 24.9% methane from enteric fermentation in livestock, and 1.1 and 16.7% non-CO_2_ GHGs from field burning and manure management, respectively (14, 15). In addition to these direct emissions, agriculture also contributes to “hidden” emissions. China's fertilizer and pesticide usage exceeds the global average by approximately three times and 2.5–5 times, respectively (16). Excessive fertilizer and pesticide use, straw burning, and residual agricultural films during crop cultivation release substantial amounts of greenhouse gases (17). When these “hidden” emissions are considered, agriculture's contribution to GHG emissions exceeds 18%. Therefore, agricultural carbon emissions represent a major source of global emissions, and their reduction is a critical and indispensable step toward achieving carbon peaking and carbon neutrality goals (18).

Green agricultural production represents the primary approach for promoting agricultural carbon neutrality (19). The concept of “sustainable development,” formally introduced by Mrs. Brundtland, the former Prime Minister of Norway, in 1987, serves as the origin and guiding ideology for green production and its application to agriculture (20). Arable land, as a crucial carrier of agricultural production, functions both as a significant carbon reservoir and a carbon sequestration unit. Implementing conservation tillage is an effective means of advancing agricultural carbon neutrality (21). However, the progress of arable land conservation in China has been relatively slow (22). The application of highly toxic chemical fertilizers and pesticides (23), the loss of soil organic matter (24), the decline in humus content, and the inefficient utilization of high-quality arable land are prevalent, resulting in a continuous degradation of arable land quality (25, 26). Agricultural green production plays a crucial role in land conservation and environmental protection. The All-China Federation of Supply and Marketing Cooperatives has issued the “Green Agricultural Inputs Upgrade Action Plan (2022–2025).” Leveraging the initiative to establish demonstration zones for reduced fertilizer use and increased efficiency, the Chinese government has adopted a series of technical models involving soil improvement, soil fertility enhancement, governance and restoration, and fertilizer reduction and efficiency gains. These measures aim to guide and encourage agricultural producers to adopt controlled-release fertilizers, water-soluble fertilizers, bio-fertilizers, and new types of fertilizers, while increasing the application of organic fertilizers and reducing irrational fertilizer inputs. Agricultural input enterprises must firmly uphold the concept that “lucid waters and lush mountains are invaluable assets,” intensify research, production, and sales of green agricultural inputs, and expand the procurement and supply of novel efficiency-enhancing fertilizers and high-efficiency, low-toxicity, low-residue pesticides. The Ministry of Agriculture and Rural Affairs has issued the “Guiding Opinions on Promoting the Construction of Ecological Farms,” which serves as an effective practice to enhance agricultural quality, effectiveness, and competitiveness, and as a powerful measure to advance agricultural green development.

It is evident that the government is a strong proponent of green production, with policy formulation and implementation playing a vital role in guiding and regulating agricultural input enterprises and farm operations. Scientific and reasonable incentive and penalty policies can promote resource emission reduction by agricultural enterprises and farms, facilitating optimal resource allocation. As intermediaries in this tripartite game, agricultural input enterprises should actively respond to government decisions by engaging in research, production, and sales of low-carbon agricultural inputs. Meanwhile, farms—being the direct implementers of agricultural production—must consider national policy requirements while balancing their own interests when deciding whether to apply low-carbon agricultural inputs. Therefore, the joint behavior and decision-making of multiple stakeholders involved in agricultural green production collectively influence the realization of agricultural carbon neutrality.

Current research on the behavioral decision-making of stakeholders in agricultural green production leverages a variety of approaches. Classical game theory has been extensively applied in exploring the decision-making processes and game mechanisms of multiple interested parties. Shi et al. (27) analyzed, from a game-theoretic perspective, the causes of food quality and safety issues in the agricultural product supply chain and the influence of government incentives and regulatory mechanisms on the decision-making of farmers and food producers. It was argued that the government should provide subsidies to farmers to encourage them to cultivate high-quality green crops, thereby ensuring the safety of the food source (28). Cohen et al. (29) analyzed the impact of government green-technology subsidies on manufacturing and consumer decisions based on a two-stage Stackelberg game that incorporated the government, manufacturers, and consumers within the same game system (30). Asakura et al. (31) employed LC-MS/MS to determine the effects of agricultural products and enazolol in livestock products. They concluded that government actions, such as formulating regulations and policies related to green agricultural development and increasing subsidies for farmers' green production behaviors, can promote farmers' adoption of green production behaviors in agricultural production activities and emphasize sustainable agricultural development (32). Song et al. (33) constructed a government-manufacturer-farmer model to refine and quantify risk behaviors in the process of supply-chain risk management. The study concluded that the higher the food price, the greater the penalty needed to deter the use of fertilizers and pesticides (27). Cui et al. (34) established an evolutionary game model between the government and farmers, as well as between farmers and agribusinesses, to identify the optimal stabilization strategy for the dissemination of green technology in agriculture (29). Kang et al. (35) applied evolutionary game theory to the field of carbon neutrality, studying the optimization of low-carbon supply-chain firm behaviors and strategic issues related to government low-carbon policies and emerging low-carbon markets. They concluded that governments can encourage firms to reduce carbon emissions by controlling carbon prices rather than imposing carbon caps (31). Tian et al. (36) used evolutionary game analysis to construct a tripartite evolutionary model of the government, farmers, and consumers, aiming to promote the reduction of fertilizer application in agriculture. It was found that increasing the government's ecological compensation for farmers to reduce fertilizer application to 200%−267% of the original level could motivate farmers to reduce fertilizer use (33). Teng and Lin (37) constructed a tripartite evolutionary game model of local governments, agricultural service organizations, and farmers to explore the behavioral decisions of stakeholders involved in arable land conservation. The conclusion was that increasing farmers' fines could be implemented as a long-term arable land conservation policy across the Northeast region (34). Although agricultural carbon neutrality is a crucial concern, existing studies mainly focus on the two-sided games of relevant stakeholders or do not consider agricultural enterprises as relevant stakeholders. Moreover, no study has been conducted on agricultural green production using game-theoretic methods to incorporate the government, agricultural enterprises, and farms into the same game system, and carbon-neutral subsidies and carbon-tax rebates have not been introduced into relevant research. Evidently, the research on the decision-making of interest actors related to agricultural green production is at a preliminary stage.

**Figure 1:**
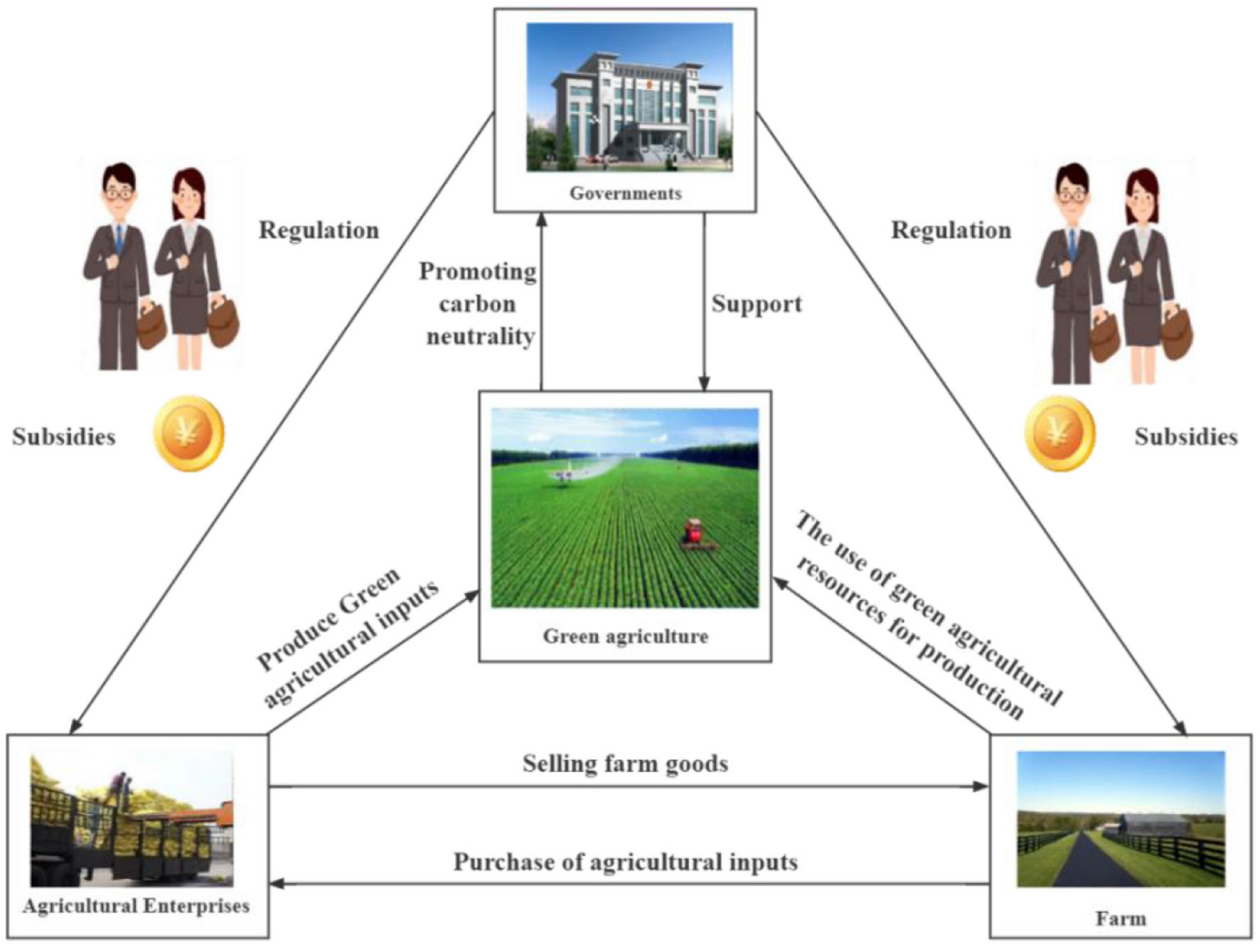
Dynamic interactions among stakeholders under green production in rural areas.

**Figure 2:**
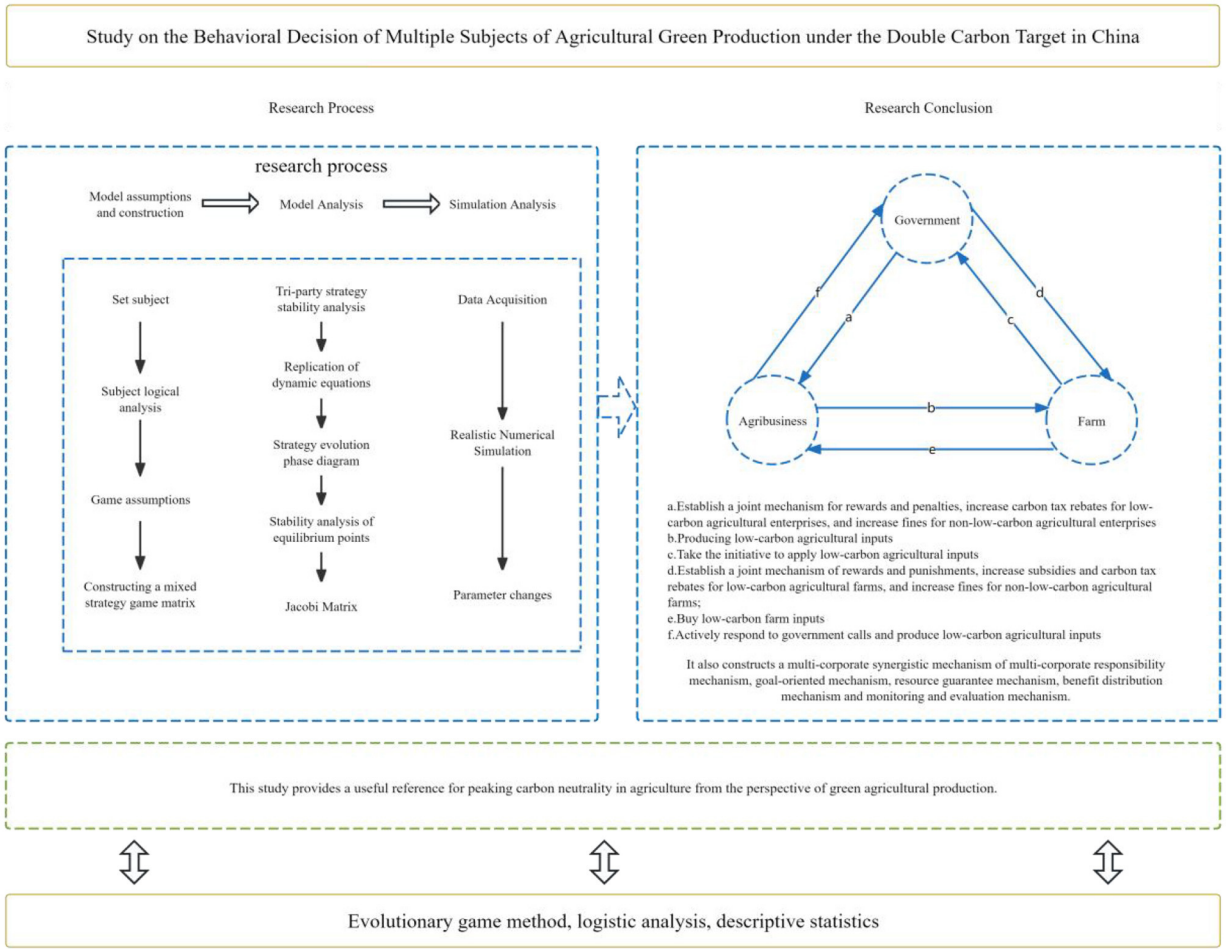
Chart summary.

In summary, numerous scholars have systematically investigated the behavioral decisions of stakeholders associated with agricultural green production, furnishing abundant references for subsequent research. This study constructs a tripartite evolutionary game model with the government, agricultural enterprises, and farms as decision-making entities under the dual-carbon objective. It innovatively incorporates two parameters, namely a carbon tax and a carbon-tax rebate, into the game model to explore the feasibility of their implementation. Moreover, it unveils the evolutionary mechanism of the decision-making behaviors of the three-party game players in agricultural green production under the dual-carbon objective in China and depicts the dynamic interactions among stakeholders, as illustrated in Figure 1. The study comprehensively considers the influence of agricultural green production factors on the carbon-peaking and carbon-neutrality goals. By altering the parameter values, it identifies the equilibrium and stability conditions for the three stakeholders' decisions to reach the ideal state. This aims to offer valuable references for the government, agricultural enterprises, and farm owners to engage in green production behaviors, thereby providing an effective approach for China to achieve its carbon-peaking and carbon-neutrality goals. The chart summary is as illustrated in Figure 2.

## 2 Triangular evolutionary game model assumptions

Hypothesis 1: Basic Assumptions of the Tripartite Game Model. The tripartite game model posits an asymmetric interaction among stakeholders, each operating under conditions of bounded rationality and seeking to maximize their individual utility. Within this framework, the “government” is specifically defined as county-level administrations responsible for policy formulation, regulatory oversight, and resource allocation. “Agricultural input enterprises” are those entities engaged in the production of pesticides and fertilizers, whose outputs directly influence the sustainability of agricultural practices. “Farms” are modeled as boundedly rational actors whose decisions are shaped by external environmental factors, policy incentives, and self-interest. The model's asymmetry stems from the divergent objective functions and constraints faced by each stakeholder. The government aims to maximize overall social and environmental welfare, agricultural input enterprises prioritize profit maximization, and farms primarily focus on their individual economic gains. This inherent asymmetry, while introducing complexity, enhances the model's ecological validity by reflecting the multifaceted dynamics of real-world agricultural systems.

Hypothesis 2: The Role and Behavioral Logic of the Government. As the primary regulatory body overseeing green agricultural production, the government functions as a steward of the broader environment. Its decision-making processes extend beyond purely economic considerations to incorporate factors such as rural development, societal impacts, government performance (38, 39), regulatory costs, carbon emissions, and grain production. The government's strategic options within the game include the implementation of policy instruments, such as fines and subsidies, designed to incentivize sustainable agricultural practices. Critically, the government's regulatory behavior is not static; it adapts to prevailing conditions. When regulatory costs become excessively high or when significant progress in green agricultural production has been achieved, the government may gradually reduce regulatory intensity or even transition to a non-regulatory posture. This adaptive behavior reflects the need to balance regulatory effectiveness with resource constraints. Furthermore, the government's policy decisions are influenced by evaluations from higher-level authorities and public opinion, further complicating its decision-making calculus.

Hypothesis 3: The Role and Behavioral Logic of Agricultural Input Enterprises. Agricultural input enterprises, responsible for producing essential agricultural materials such as fertilizers and pesticides, exert substantial influence on the sustainability of farming practices. Their strategic choices within the model are contingent upon government reward and punishment policies, production costs, and anticipated profits. Enterprises prioritizing short-term profitability may opt for the production of non-low-carbon agricultural inputs to minimize expenses and maximize immediate gains. Conversely, those adopting a longer-term perspective may choose to produce low-carbon alternatives to align with policy directives and bolster their corporate reputation. Market competition and technological capabilities also shape enterprise decision-making. In environments characterized by weak policy incentives or lax regulatory enforcement, enterprises are more likely to adopt cost-effective, albeit more polluting, production methods. Conversely, strong policy incentives coupled with stringent regulations encourage a transition toward more sustainable practices.

Hypothesis 4: The Role and Behavioral Logic of Farms. As the direct practitioners of agricultural production, farms' behavioral choices are shaped by a complex interplay of factors, including government incentives, profit and loss considerations, and environmental consciousness. Within the game framework, farms' strategic decisions are predominantly manifested in their selection of agricultural inputs. Farms with heightened environmental awareness are more inclined to choose low-carbon inputs to mitigate environmental impacts and enhance long-term sustainability. Conversely, those with lower environmental awareness may prioritize non-low-carbon inputs to minimize production costs and maximize short-term profits. Information access, technological capabilities, and prevailing market conditions also significantly influence farm decision-making. In scenarios where policy incentives are inadequate or information asymmetry prevails, farms are more likely to adopt low-cost, high-pollution production methods. Conversely, sufficient incentives and transparent information facilitate the adoption of green production practices.

### 2.1 Model parameters

#### 2.1.1 Government-related gains and losses are assumed to be as follows

When the government implements a regulatory decision, the associated regulatory cost is denoted as *C*_1_. The regulation yields several effects: it enhances the diet quality of green agricultural products across the population, which contributes to improved physical fitness; this benefit is quantified as *D*_1_. Additionally, government regulation positively impacts agricultural carbon neutrality, represented by *W*_1_, and bolsters the government's credibility, indicated as *F*_1_. Penalties paid by companies producing non-low-carbon agricultural products are denoted as *M*_1_. Simultaneously, the government provides subsidies of amount *M*_2_ to low-carbon agricultural enterprises, subsidies of amount *M*_3_ to farms utilizing low-carbon agricultural resources, and fines of amount *M*_4_ to farms utilizing non-low-carbon resources. The government also allocates funds of amount *M*_5_ for research and development of relevant technologies and equipment for low-carbon agricultural enterprises, and funds of amount *M*_6_ for the procurement of equipment for farms using low-carbon resources. For enterprises adopting low-carbon agricultural inputs, the carbon-tax rebate is *Q*_1_; conversely, for those using non-low-carbon inputs, an additional carbon tax *Q*_2_ is imposed. For farms applying low-carbon agricultural materials, the carbon-tax rebate is *Q*_3_, and for farms applying non-low-carbon agricultural materials, the additional carbon tax is *Q*_4_. The reduction in government revenue due to preferential interest rates on loans extended to low-carbon agricultural enterprises is denoted as *A*_1_, and the reduction due to similar interest rate concessions to farms utilizing low-carbon resources is *A*_2_. Furthermore, the decrease in revenue and the increase in expenditures resulting from reduced insurance premiums and expanded coverage for farms using low-carbon agricultural inputs are represented as *B*.

When the government opts not to regulate, the monitoring cost savings are *C*_1_, the negative impact on food security stemming from the long-term application of non-low-carbon pesticides and fertilizers—leading to soil degradation—is represented as *D*_2_. The negative environmental effect of abstaining from regulation, particularly in terms of hindering progress toward agricultural carbon neutrality, is denoted as *W*_2_. The decline in the government's credibility due to inaction is indicated as *F*_2_. The government bears costs related to environmental remediation, represented as *P*. Additionally, the decision not to regulate may be influenced by a slowdown in mitigating negative international impacts associated with achieving carbon neutrality, exemplified by China's pledge in September 2020 to reach carbon peaking by 2030 and carbon neutrality by 2060, which is denoted as *S*.

#### 2.1.2. The relevant profit and loss assumptions for agribusiness are as follows

When an enterprise adopts the production of low-carbon agricultural materials, the basic operational cost is denoted as *C*_2_. The additional costs, such as research and development efforts and equipment upgrades, are represented as *C*_3_. The additional profit resulting from this shift is *C*_5_. The enterprise is offered preferential pricing *U* for agricultural materials used in low-carbon farms. Furthermore, the government provides subsidies of amount *M*_2_ to low-carbon agricultural enterprises, along with funding of *M*_5_ for research, technological development, and equipment acquisition. The government also grants a carbon tax rebate of *Q*_1_ to enterprises producing low-carbon agricultural products. These measures have a favorable impact on the long-term development prospects of the enterprise, denoted as *H*_1_, and contribute to reputation enhancement *L*_1_ (easy to become a model low-carbon enterprises set up by the state, improve social recognition), the government gives the enterprises that adopt low-carbon agricultural production loan interest rate preferential *A*_1_.

Conversely, if an enterprise produces non-low-carbon agricultural materials, it incurs the basic operational cost *C*_2_, but benefits from savings on additional costs *C*_3_. However, it must pay an increased carbon tax *Q*_2_. Penalties paid to the government due to non-compliance are represented as *M*_1_. Such practices negatively impact the enterprise's long-term development, denoted as *H*_2_, and harm its reputation *L*_2_.

#### 2.1.3. Farm-related gains and losses are assumed to be as follows

When farms utilize low-carbon farming materials, they incur the basic operational cost *C*_4_, along with additional costs *C*_5_ for fertilizers and pesticides, and *C*_6_ for the introduction of new equipment. Such practices facilitate the development of long-term, trust-based partnerships *Z* between low-carbon farming enterprises and farms, fostering mutual cooperation and shared growth. Farms also benefit from preferential pricing *U* on farming materials, quality assurance *V*, and acquisition of low-carbon farming equipment *M*_6_. Additionally, farms receive a carbon tax rebate *Q*_3_, which enhances the land's carbon neutrality *T*_1_, boosts crop growth through improved soil fertility, and thereby increases the crops' carbon sink and sequestration capacity *K*. These practices generate extra income from higher-quality agricultural products *N*, positively impact the farm's long-term development prospects *H*_3_, and enhance the farm's reputation *L*_3_, positioning it as a model low-carbon farm recognized nationally. Government subsidies for farms employing low-carbon resources *M*_3_, preferential loan interest rates *A*_2_, and expanded scope and amounts of government agricultural insurance claims *B* further support sustainable development.

Conversely, if farms use non-low-carbon farming materials, they pay the basic operational cost *C*_4_ and incur an additional carbon tax *Q*_4_. Such practices diminish the farm's carbon neutrality *T*_2_, and reduce the benefits obtained from lower cultivation costs *C*_5_ and *C*_6_. These behaviors negatively affect the farm's long-term development *H*_4_ and impair its reputation *L*_4_. Moreover, the government enforces fines *M*_4_ on farms utilizing non-low-carbon materials as a punitive measure.

Based on the above assumptions, this paper starts from the different strategies of each subject, which can be obtained with eight strategy combinations, which can be used to construct a three-way game gain-loss matrix based on the gain combination, as shown in Table 1.

**Table 1:** Profit and loss combinations for the three-party evolutionary game.

**Strategy portfolio**	**Government benefits**	**Agribusiness revenue**	**Farm income**
Regulatory, low carbon, low carbon	$\begin{array}{c} D_{1}+W_{1}+F_{1}-C_{1}\\ -M_{2}-M_{3}-M_{5}-M_{6}\\ -Q_{1}-Q_{3}-A_{1}-A_{2}-B \end{array}$	$\begin{array}{c} Z+U+V+M_{6}+Q_{3}+T_{1}\\ +K+N+H_{3}+L_{3}+M_{3}\\ +A_{2}+B-C_{4}-C_{5}-C_{6} \end{array}$	$\begin{array}{c} Z+U+V+M_{6}+Q_{3}+T_{1}\\ +K+N+H_{3}+L_{3}+M_{3}\\ +A_{2}+B-C_{4}-C_{5}-C_{6} \end{array}$
Regulatory, low-carbon, non-low-carbon	$\begin{array}{c} F_{1}+M_{4}+Q_{4}-C_{1}\\ -M_{2}-M_{5}-Q_{1}-A_{1} \end{array}$	$\begin{array}{c} M_{2}+M_{5}+Q_{1}+H_{1}\\ +L_{1}+A_{1}-C_{2}-C_{3} \end{array}$	* $\begin{array}{c} C_{6}+C_{5}-C_{4}-Q_{4}\\ -T_{2}-H_{4}-L_{4}-M_{4} \end{array}$ *
Regulatory, non-low carbon, low carbon	$\begin{array}{c} D_{1}+W_{1}+F_{1}+\\ M_{1}+Q_{2}-C_{1}-M_{3}\\ -M_{6}-Q_{3}-A_{2}-B \end{array}$	*C*_3_−*C*_2_−*Q*_2_−*M*_1_−*H*_2_−*L*_2_	$\begin{array}{c} M_{6}+Q_{3}+T_{1}+K+N+H_{3}+L_{3}\\ +M_{3}+A_{2}+B-C_{4}-C_{5}-C_{6} \end{array}$
Regulatory, Non-Low Carbon, Non-Low Carbon	*M*_1_+*M*_4_+*Q*_2_+*Q*_4_−*C*_1_	*C*_3_−*C*_2_−*Q*_2_−*M*_1_−*H*_2_−*L*_2_	$\begin{array}{c} C_{6}+C_{5}-C_{4}-Q_{4}\\ -T_{2}-H_{4}-L_{4}-M_{4} \end{array}$
Unregulated, low-carbon, low-carbon	**C*_1_−*F*_2_*	*_*C*_5_−*C*_2_−*C*_3_−*U*_*	$\begin{array}{c} Z+U+V+T_{1}+K\\ +N-C_{4}-C_{5}-C_{6} \end{array}$
Unregulated, low-carbon, non-low-carbon	*C*_1_−*D*_2_−*W*_2_−*F*_2_−*P*−*S*	*_−_*C*__2_−*C*_3__*	$\begin{array}{c} C_{6}+C_{5}-C_{4}\\ -T_{2}-H_{4}-L_{4} \end{array}$
Unregulated, non-low carbon, low carbon	**C*_1_−*F*_2_*	*_*C*_3_−*C*_2_−*H*_2_−*L*_2__*	*T*_1_+*K*+*N*−*C*_4_−*C*_5_−*C*_6_
Non-regulatory, non-low carbon, non-low carbon	**C*_1_−*D*_2_−*W*_2_−*F*_2_−*P*−*S**	*_*C*_3_−*C*_2_−*H*_2_−*L*_2__*	$\begin{array}{c} C_{6}+C_{5}-C_{4}\\ -T_{2}-H_{4}-L_{4} \end{array}$

## 3 Equilibrium analysis of the three-party evolutionary game model

Assume that in the initial stage of the game, the proportion of government adopting regulatory strategy is *x*, the proportion of adopting non-regulatory strategy is 1-*x*, the proportion of agribusiness adopting low-carbon agribusiness is *y*, the proportion of agribusiness adopting non-low-carbon agribusiness is 1-*y*, the proportion of farms applying low-carbon agribusiness is *z*, and the proportion of farms applying non-low-carbon agribusiness is 1-*z*.

1. The expected benefits of the government's decision to “regulate” and “not to regulate” are *V*_1__*X*_, *V*_2__*X*_and the average benefits of $\bar{V_{X}}$, respectively:

(1) $$\begin{array}{l} {\text{V}}_{\text{1}X}=y(M_{1}-M_{2}-M_{5}-Q_{1}-A_{1}-Q_{2})+z(D_{1}+W_{1}\\ \;-M_{3}-M_{6}-Q_{3}-A_{2}-B-M_{4}-Q_{4})+(M_{1}+M_{4}\\ \;+Q_{2}+Q_{4}-C_{1}) \end{array}$$

(2) $$\begin{array}{l} V_{2X}=z\left(-D_{2}-W_{2}-P-S\right)+C_{1}-D_{2}-W_{2}-F_{2}-P-S \end{array}$$

(3) $$\begin{array}{l} \bar{V_{X}}=x\cdot V_{1X}+\left(1-x\right)V_{2X} \end{array}$$

2. The expected benefits of the agribusiness decision to “adopt low-carbon agribusiness” and “adopt non-low-carbon agribusiness” are *V*_1__*Y*_, *V*_2__*Y*_ and the average benefits of $\bar{V_{Y}}$, respectively:

(4) $$\begin{array}{l} V_{1Y}=x\left(M_{2}+M_{5}+Q_{1}+H_{1}+L_{1}+A_{1}\right)+z\left(C_{5}-U\right)-C_{2}-C_{3} \end{array}$$

(5) $$\begin{array}{l} V_{2Y}=x\left(-Q_{2}-M_{1}\right)+C_{3}-C_{2}-H_{2}-L_{2} \end{array}$$

(6) $$\begin{array}{l} \bar{V_{Y}}=y\cdot V_{1Y}+\left(1-y\right)V_{2Y} \end{array}$$

3. The expected benefits of the “low-carbon farming” and “non-low-carbon farming” decisions are *V*_1*Z*_, *V*_2*Z*_, and the average benefits of $\bar{V_{Z}}$, respectively:

(7) $$\begin{array}{l} V_{1Z}=x(M_{6}+Q_{3}+3H_{3}+L_{3}+M_{3}+A_{2}+B)\\ \;+y(Z+U+V)+T_{1}+K+N-C_{4}-C_{5}-C_{6} \end{array}$$

(8) $$\begin{array}{l} V_{2Z}=x\left(-Q_{4}-M_{4}\right)+C_{6}+C_{5}-C_{4}-T_{2}-H_{4}-L_{4} \end{array}$$

(9) $$\begin{array}{l} \bar{V_{Z}}=z\cdot V_{1Z}+\left(1-z\right)V_{2Z} \end{array}$$

### 3.1 Government replication dynamic equation analysis

The government regulatory decision replication dynamic equation is:


(10) $$\begin{array}{l} F\left(x\right)=\frac{dx}{dt}=x\left(V_{1X}-\bar{V_{X}}\right) \end{array}$$

1. When$y=\frac{-z\left(D_{1}+W_{1}+F_{1}+D_{2}+W_{2}+P+S-M_{3}-M_{6}-Q_{3}-A_{2}-B-M_{4}-Q_{4}\right)-\left(M_{1}+M_{4}+Q_{2}+Q_{4}+D_{2}+W_{2}+F_{2}+P+S-2C_{1}\right)}{-zF_{1}+F_{1}+M_{1}-M_{2}-M_{5}-Q_{1}-A_{1}-Q_{2}}$, then *F*(*x*) = 0 is obtained, which means that whether the government imposes regulation is a steady state.
2. When$y\neq \frac{-z\left(D_{1}+W_{1}+F_{1}+D_{2}+W_{2}+P+S-M_{3}-M_{6}-Q_{3}-A_{2}-B-M_{4}-Q_{4}\right)-\left(M_{1}+M_{4}+Q_{2}+Q_{4}+D_{2}+W_{2}+F_{2}+P+S-2C_{1}\right)}{-zF_{1}+F_{1}+M_{1}-M_{2}-M_{5}-Q_{1}-A_{1}-Q_{2}}$, let *F*(*x*) = 0 be obtained, *x* = 0, *x* = 1 may be the evolutionary stability point. From the stability theorem of replicated dynamic equations, *x* as a fixed strategy needs to meet *F*(*x*) = 0 and *F'*(*x*) < 0.

Derivation of *F*(*x*) yields:


(11) $$F^{\prime }(x)=(1-2x)\left[\begin{array}{l} y(M_{1}-M_{2}-M_{5}-Q_{1}-A_{1}-Q_{2})+z(D_{1}+W_{1}+D_{2}+W_{2}+P+S-M_{3}-M_{6}\\ -Q_{3}-A_{2}-B-M_{4}-Q_{4})+M_{1}+M_{4}+Q_{2}+Q_{4}+D_{2}+W_{2}+F_{2}+P+S-2C_{1} \end{array}\right]$$

3. When$y>\frac{-z\left(D_{1}+W_{1}+F_{1}+D_{2}+W_{2}+P+S-M_{3}-M_{6}-Q_{3}-A_{2}-B-M_{4}-Q_{4}\right)-\left(M_{1}+M_{4}+Q_{2}+Q_{4}+D_{2}+W_{2}+F_{2}+P+S-2C_{1}\right)}{-zF_{1}+F_{1}+M_{1}-M_{2}-M_{5}-Q_{1}-A_{1}-Q_{2}}$, then $\frac{dF\left(x\right)}{dx}{|}_{x=0}>0$, $\frac{dF\left(x\right)}{dx}{|}_{x=1}<0$. Therefore, *x =* 1 is the evolutionary stability point.
4. When$y<\frac{-z\left(D_{1}+W_{1}+F_{1}+D_{2}+W_{2}+P+S-M_{3}-M_{6}-Q_{3}-A_{2}-B-M_{4}-Q_{4}\right)-\left(M_{1}+M_{4}+Q_{2}+Q_{4}+D_{2}+W_{2}+F_{2}+P+S-2C_{1}\right)}{-zF_{1}+F_{1}+M_{1}-M_{2}-M_{5}-Q_{1}-A_{1}-Q_{2}}$, then $\frac{dF\left(x\right)}{dx}{|}_{x=0}<0$, $\frac{dF\left(x\right)}{dx}{|}_{x=1}>0$. Therefore, *x* = 0 is the evolutionary stability point.

### 3.2 Agribusiness replication dynamic equation analysis

Agribusinesses adopt the replication dynamic equation for producing low-carbon agribusiness decisions as


(12) $$\begin{array}{l} F\left(y\right)=\frac{dy}{dz}=y\left(V_{1Y}-\bar{V_{Y}}\right) \end{array}$$

When $x=\frac{-z\left(C_{5}-U\right)-\left(H_{2}+L_{2}-2C_{3}\right)}{M_{1}+M_{2}+M_{5}+Q_{1}+Q_{2}+H_{1}+L_{1}+A_{1}}$, *F*(*y*) *=* 0 is obtained, which means that agribusinesses adopt to produce low-carbon agribusiness or adopt to produce non-low-carbon agribusiness are steady state.

When $x\neq \frac{-z\left(C_{5}-U\right)-\left(H_{2}+L_{2}-2C_{3}\right)}{M_{1}+M_{2}+M_{5}+Q_{1}+Q_{2}+H_{1}+L_{1}+A_{1}}$, let *F*(*y*) *=* 0 be obtained, *y* = 0, *y* = 1 may be the evolutionary stabilization point. From the stability theorem of replicated dynamic equations, *y* as a stabilization strategy needs to meet *F*(*y*) = 0 and *F'*(*y*) < 0.

Derivation of *F*(*y*) yields:


(13) $$F^{\prime }(y)=\frac{dF(y)}{dy}=(1-2y)\left[\begin{array}{l} x(M_{1}+M_{2}+M_{5}+Q_{1}+Q_{2}+H_{1}+L_{1}+A_{1})\\ +z(C_{5}-U)+H_{2}+L_{2}-2C_{3} \end{array}\right]$$

When, $x>\frac{-z\left(C_{5}-U\right)-\left(H_{2}+L_{2}-2C_{3}\right)}{M_{1}+M_{2}+M_{5}+Q_{1}+Q_{2}+H_{1}+L_{1}+A_{1}}$, $\frac{dF\left(y\right)}{dy}{|}_{y=0}>0$, $\frac{dF\left(y\right)}{dy}{|}_{y=1}<0$. Therefore, *y* = 1 is the evolutionary stability point.

When, $x<\frac{-z\left(C_{5}-U\right)-\left(H_{2}+L_{2}-2C_{3}\right)}{M_{1}+M_{2}+M_{5}+Q_{1}+Q_{2}+H_{1}+L_{1}+A_{1}}$, $\frac{dF\left(y\right)}{dy}{|}_{y=0}<0$, $\frac{dF\left(y\right)}{dy}{|}_{y=1}>0$. Therefore, *y* = 0 is the evolutionary stability point.

### 3.3 Farm replication dynamic equation analysis

The replicated dynamic equation for the decision replication of low-carbon farm application is:


(14) $$\begin{array}{l} F\left(z\right)=\frac{dz}{dt}=z\left(V_{1z}-\bar{V_{z}}\right) \end{array}$$

1. When $y=\frac{-x\left(M_{4}+M_{6}+Q_{3}+Q_{4}+3H_{3}+L_{3}+M_{3}+A_{2}+B_{1}\right)-\left(T_{1}+T_{2}+H_{4}+L_{4}+K+N-2C_{5}-2C_{6}\right)}{Z+U+V}$, *F*(*z*) = 0 is obtained, which means that whether the farm applies low carbon farming is a steady state.
2. When $y\neq \frac{-x\left(M_{4}+M_{6}+Q_{3}+Q_{4}+3H_{3}+L_{3}+M_{3}+A_{2}+B_{1}\right)-\left(T_{1}+T_{2}+H_{4}+L_{4}+K+N-2C_{5}-2C_{6}\right)}{Z+U+V}$, let *F*(*z*) = 0, *z* = 0, *z* = 1 be the possible evolutionary stability points. By the stability theorem of replicated dynamic equations, *z* as a stabilization strategy needs to meet *F*(*z*) = 0 and *F'*(*z*) < 0.

Derivation of *F*(*z*) yields:


(15) $$F^{\prime }(z)=\frac{dF(z)}{dz}=(1-2z)\left[\begin{array}{l} x(M_{4}+M_{6}+Q_{3}+Q_{4}+3H_{3}+L_{3}+M_{3}+A_{2}+B_{1})\\ +y(Z+U+V)+T_{1}+T_{2}+H_{4}+L_{4}+K+N-2C_{5}-2C_{6} \end{array}\right]$$

3. When $y>\frac{-x\left(M_{4}+M_{6}+Q_{3}+Q_{4}+3H_{3}+L_{3}+M_{3}+A_{2}+B_{1}\right)-\left(T_{1}+T_{2}+H_{4}+L_{4}+K+N-2C_{5}-2C_{6}\right)}{Z+U+V}$

$\frac{dF\left(z\right)}{dz}{|}_{z=0}>0$, $\frac{dF\left(z\right)}{dz}{|}_{z=1}<0$, so *z* = 1 is the evolutionary stability point.

4. When $y<\frac{-x\left(M_{4}+M_{6}+Q_{3}+Q_{4}+3H_{3}+L_{3}+M_{3}+A_{2}+B_{1}\right)-\left(T_{1}+T_{2}+H_{4}+L_{4}+K+N-2C_{5}-2C_{6}\right)}{Z+U+V}$

$\frac{dF\left(z\right)}{dz}{|}_{z=0}<0$, $\frac{dF\left(z\right)}{dz}{|}_{z=1}>0$, so *z* = 0 is the evolutionary stability point.

### 3.4 Evolutionary stability analysis

From Equations 10, 12, 14, it can be discerned that the government's regulatory decision is associated with the agribusinesses' choice of adopting low-carbon farming practices. The agribusinesses' decision to embrace low-carbon farming is contingent upon both the government's regulatory decision and the farms' decision to apply low-carbon farming methods. Moreover, the farms' decision to utilize low-carbon farming materials is related to the agribusinesses' decision to produce low-carbon farming inputs. In light of these relationships, this study conducts a step-by-step analysis of the strategic evolutionary stability of three stakeholders. Specifically, it performs an evolutionary stability analysis between the government and agribusinesses, as well as between agribusinesses and farms.

(1) Analysis of the evolutionary stability of government and agribusiness

From Equations 10, 12, we can see that the dynamic game between the government and agribusiness contains five equilibria (0, 0), (0, 1), (1, 0), (1, 1), and ($x^{*}=\frac{-z\left(C_{5}-U\right)-\left(H_{2}+L_{2}-2C_{3}\right)}{M_{1}+M_{3}+M_{5}+Q_{1}+Q_{2}+H_{1}+L_{1}+A_{1}}$, $y^{*}=\frac{-z\left(D_{1}+W_{1}+F_{1}+D_{2}+W_{2}+P+S-M_{3}-M_{6}-Q_{3}-A_{2}-B-M_{4}-Q_{4}\right)-\left(M_{1}+M_{4}+Q_{2}+Q_{4}+D_{2}+W_{2}+F_{2}+P+S-2C_{1}\right)}{-zF_{1}+F_{1}+M_{1}-M_{2}-M_{5}-Q_{1}-A_{1}-Q_{2}}$). The game holds when and only when $0\leq \frac{-z\left(C_{5}-U\right)-\left(H_{2}+L_{2}-2C_{3}\right)}{M_{1}+M_{2}+M_{5}+Q_{1}+Q_{2}+H_{1}+L_{1}+A_{1}}\leq 1$, $0\leq \frac{-z\left(D_{1}+W_{1}+F_{1}+D_{2}+W_{2}+P+S-M_{3}-M_{6}-Q_{3}-A_{2}-B-M_{4}-Q_{4}\right)-\left(M_{1}+M_{4}+Q_{2}+Q_{4}+D_{2}+W_{2}+F_{2}+P+S-2C_{1}\right)}{-zF_{1}+F_{1}+M_{1}-M_{2}-M_{5}-Q_{1}-A_{1}-Q_{2}}\leq 1$

Jacobi matrix:


$$J_{1}=\left[\begin{array}{l} (1-2x)\left[\begin{array}{l} y(M_{1}-M_{2}-M_{5}-Q_{1}-A_{1}-Q_{2})\\ +z(D_{1}+W_{1}+D_{2}+W_{2}+P+S-M_{3}-M_{6}-Q_{3}-A_{2}-B-M_{4}-Q_{4})\\ +M_{1}+M_{4}+Q_{2}+Q_{4}+D_{2}+W_{2}+P+S-2C_{1}) \end{array}\right],x(1-x)\left[-zF_{1}+F_{1}+M_{1}-M_{2}-M_{5}-Q_{1}-A_{1}-Q_{2}\right]\\ y(1-y)(M_{1}+M_{2}+M_{5}+Q_{1}+Q_{2}+H_{1}+L_{1}+A_{1}),(1-2y)\left[x(M_{1}+M_{2}+M_{5}+Q_{1}+Q_{2}+H_{1}+L_{1}+A_{1})+z(C_{5}-U)+H_{2}+L_{2}-2C_{3}\right] \end{array}\right]$$

The matrix *J*_1_ rows and columns:


$$\begin{array}{l} \det J_{1}=(1-2x)\left[\begin{array}{l} y(M_{1}-M_{2}-M_{5}-Q_{1}-A_{1}-Q_{2})+z(D_{1}+W_{1}+D_{2}+W_{2}+P\\ +S-M_{3}-M_{6}-Q_{3}-A_{2}-B-M_{4}-Q_{4})+M_{1}+M_{4}+Q_{2}+Q_{4}+D_{2}+W_{2}+P+S-2C_{1}) \end{array}\right]\\ (1-2y)\left[x(M_{1}+M_{2}+M_{5}+Q_{1}+Q_{2}+H_{1}+L_{1}+A_{1})+z(C_{5}-U)+H_{2}+L_{2}-2C_{3}\right]\\ -x(1-x)[-zF_{1}+F_{1}+M_{1}-M_{2}-M_{5}-Q_{1}-A_{1}-Q_{2}]y(1-y)(M_{1}+M_{2}+M_{5}+Q_{1}+Q_{2}+H_{1}+L_{1}+A_{1}) \end{array}$$

Matrix *J*_1_signs:


$$\begin{array}{l} \text{tr}J_{1}=(1-2x)\left[\begin{array}{l} -yzF_{1}+y(F_{1}+M_{1}-M_{2}-M_{5}-Q_{1}-A_{1}-Q_{2})\\ +z(D_{1}+W_{1}+F_{1}+D_{2}+W_{2}+P+S-M_{3}-M_{6}-Q_{3}-A_{2}-B-M_{4}-Q_{4})\\ +M_{1}+M_{4}+Q_{2}+Q_{4}+D_{2}+W_{2}+P+S-2C_{1}) \end{array}\right]\\ +(1-2y)\left[x(M_{1}+M_{2}+M_{5}+Q_{1}+Q_{2}+H_{1}+L_{1}+A_{1})+z(C_{5}-U)+H_{2}+L_{2}-2C_{3}\right] \end{array}$$

The local stability analysis was performed based on the above five equilibrium points, and the results are shown in Table 2.

**Table 2:** Analysis of the stability results of the evolutionary game between government and agribusiness.

**Balancing point**	***detJ_1_* Symbols**	***trJ_1_* Symbols**	**Results**	**Stable conditions**
*x* = 0, *y* = 0	+	−	*ESS*	$\begin{array}{c} z\left(D_{1}+W_{1}+F_{1}+D_{2}+W_{2}+P+S\right)+M_{1}+M_{4}+Q_{2}+Q_{4}+D_{2}+W_{2}+P+S<\\ z\left(M_{3}+M_{6}+Q_{3}+A_{2}+B+M_{4}+Q_{4}\right)+2C_{1} \end{array}$
				*zC*_5_+*H*_2_+*L*_2_ < 2*C*_3_+*zU*
*x* = 0, *y* = 1	+	−	*ESS*	$\begin{array}{c} z\left(D_{1}+W_{1}+F_{1}+D_{2}+W_{2}+P+S\right)+F_{1}+2M_{1}+M_{4}+Q_{2}+Q_{4}+D_{2}+W_{2}+P+S\\ <z\left(M_{3}+M_{6}+Q_{3}+A_{2}+B+M_{4}+Q_{4}+F_{1}\right)+M_{2}+M_{5}+Q_{1}+A_{1}+Q_{2}+2C_{1} \end{array}$
				*zU*+2*C*_3_<*zC*_5_+*H*_2_+*L*_2_
*x* = 1, *y* = 0	+	−	*ESS*	$\begin{array}{c} z\left(M_{3}+M_{6}+Q_{3}+A_{2}+B+M_{4}+Q_{4}\right)+2C_{1}<\\ z\left(D_{1}+W_{1}+F_{1}+D_{2}+W_{2}+P+S\right)+M_{1}+M_{4}+Q_{2}+Q_{4}+D_{2}+W_{2}+P+S \end{array}$
				*zC*_5_+*M*_1_+*M*_2_+*M*_5_+*Q*_1_+*Q*_2_+*H*_1_+*L*_1_+*A*_1_+*H*_2_+*L*_2_ < 2*C*_3_+*zU*
*x* = 1, *y* = 1	+	−	*ESS*	$\begin{array}{c} z\left(M_{3}+M_{6}+Q_{3}+A_{2}+B+M_{4}+Q_{4}+R+F_{1}\right)+M_{2}+M_{5}+Q_{1}+A_{1}+Q_{2}+2C_{1}<\\ z\left(D_{1}+W_{1}+F_{1}+D_{2}+W_{2}+P+S\right)+R+F_{1}+2M_{1}+M_{4}+Q_{2}+Q_{4}+D_{2}+W_{2}+P+S \end{array}$
				2*C*_3_+*zU*<*zC*_5_+*M*_1_+*M*_2_+*M*_5_+*Q*_1_+*Q*_2_+*H*_1_+*L*_1_+*A*_1_+*H*_2_+*L*_2_
*x* = x^*^, *y* = y^*^	0	0	Saddle point	Saddle point under any condition

(2) Analysis of the evolutionary stability of agribusiness and farms

From Equations 12, 14, we can see that the dynamic game between agribusiness and farm contains five equilibria (0, 0), (0, 1), (1, 0), (1, 1), and (${\text{y}}^{**}=\frac{-z\left(C_{5}-U\right)-\left(H_{2}+L_{2}-2C_{3}\right)}{M_{1}+M_{2}+M_{5}+Q_{1}+Q_{2}+H_{1}+L_{1}+A_{1}}$, $z^{**}=\frac{-x\left(M_{4}+M_{6}+Q_{3}+Q_{4}+3H_{3}+L_{3}+M_{3}+A_{2}+B_{1}\right)-\left(T_{1}+T_{2}+H_{4}+L_{4}+K+N-2C_{5}-2C_{6}\right)}{Z+U+V}$), which hold if and only if $0<\frac{-z\left(C_{5}-U\right)-\left(H_{2}+L_{2}-2C_{3}\right)}{M_{1}+M_{2}+M_{5}+Q_{1}+Q_{2}+H_{1}+L_{1}+A_{1}}<1$, $0<\frac{-x\left(M_{4}+M_{6}+Q_{3}+Q_{4}+3H_{3}+L_{3}+M_{3}+A_{2}+B_{1}\right)-\left(T_{1}+T_{2}+H_{4}+L_{4}+K+N-2C_{5}-2C_{6}\right)}{Z+U+V}<1$, give the dynamic game evolution related content.

Jacobi matrix: *J*_1_ =

$\left[\begin{array}{c} \left(1-2y\right)\left[x\left(M_{1}+M_{2}+M_{5}+Q_{1}+Q_{2}+H_{1}+L_{1}+A_{1}\right)+z\left(C_{5}-U\right)+H_{2}+L_{2}-2C_{3}\right],y\left(1-y\right)\left(C_{5}-U\right)\\ z\left(1-z\right)\left(Z+U+V\right),\left(1-2z\right)\left[x\left(M_{4}+M_{6}+Q_{3}+Q_{4}+3H_{3}+L_{3}+M_{3}+A_{2}+B_{1}\right)+y\left(Z+U+V\right)+T_{1}+T_{2}+H_{4}+L_{4}+K+N-2C_{5}-2C_{6}\right] \end{array}\right]$

The matrix *J*_2_rows and columns:


$$\begin{array}{l} \det J_{2}=(1-2y)\left[x(M_{1}+M_{2}+M_{5}+Q_{1}+Q_{2}+H_{1}+L_{1}+A_{1})+z(C_{5}-U)+H_{2}+L_{2}-2C_{3}\right]\\ \left(1-2z)[x(M_{4}+M_{6}+Q_{3}+Q_{4}+3H_{3}+L_{3}+M_{3}+A_{2}+B_{1})+y(Z+U+V)+T_{1}+T_{2}+H_{4}+L_{4}+K+N-2C_{5}-2C_{6}\right]\\ -y(1-y)(C_{5}-U)z(1-z)(Z+U+V) \end{array}$$

Matrix *J*_2_ signs:

$\begin{array}{c} \text{tr}J_{2}=\left(1-2y\right)\left[x\left(M_{1}+M_{2}+M_{5}+Q_{1}+Q_{2}+H_{1}+L_{1}+A_{1}\right)+z\left(C_{5}-U\right)+H_{2}+L_{2}-2C_{3}\right]+\\ \left(1-2z\right)\left[x\left(M_{4}+M_{6}+Q_{3}+Q_{4}+3H_{3}+L_{3}+M_{3}+A_{2}+B_{1}\right)+y\left(Z+U+V\right)+T_{1}+T_{2}+H_{4}+L_{4}+K+N-2C_{5}-2C_{6}\right] \end{array}$

The local stability analysis was performed based on the above five equilibrium points, and the results are shown in Table 3.

**Table 3:** Results of stability analysis of the evolutionary game of agricultural enterprises and farms.

**Balancing point**	***detJ_2_* Symbols**	***trJ_2_* Symbols**	**Results**	**Stable conditions**
*y* = 0, *z* = 0	+	−	*ESS*	*x*(*M*_1_+*M*_2_+*M*_5_+*Q*_1_+*Q*_2_+*H*_1_+*L*_1_+*A*_1_)+*H*_2_+*L*_2_ < 2*C*_3_
				*x*(*M*_4_+*M*_6_+*Q*_3_+*Q*_4_+3*H*_3_+*L*_3_+*M*_3_+*A*_2_+*B*_1_)+*T*_1_+*T*_2_+*H*_4_+*L*_4_+*K*+*N* < 2*C*_5_+2*C*_6_
*y* = 0, *z* = 1	+	−	*ESS*	*x*(*M*_1_+*M*_2_+*M*_5_+*Q*_1_+*Q*_2_+*H*_1_+*L*_1_+*A*_1_)+*C*_5_+*H*_2_+*L*_2_ < 2*C*_3_+*U*
				*x*(*M*_4_+*M*_6_+*Q*_3_+*Q*_4_+3*H*_3_+*L*_3_+*M*_3_+*A*_2_+*B*_1_)+*T*_1_+*T*_2_+*H*_4_+*L*_4_+*K*+*N*>2*C*_5_+2*C*_6_
*y* = 1, *z* = 0	+	−	*ESS*	*x*(*M*_1_+*M*_2_+*M*_5_+*Q*_1_+*Q*_2_+*H*_1_+*L*_1_+*A*_1_)+*H*_2_+*L*_2_>2*C*_3_
				*x*(*M*_4_+*M*_6_+*Q*_3_+*Q*_4_+3*H*_3_+*L*_3_+*M*_3_+*A*_2_+*B*_1_)+*Z*+*U*+*V*+*T*_1_+*T*_2_+*H*_4_+*L*_4_+*K*+*N* < 2*C*_5_+2*C*_6_
*y* = 1, *z* = 1	+	−	*ESS*	*x*(*M*_1_+*M*_2_+*M*_5_+*Q*_1_+*Q*_2_+*H*_1_+*L*_1_+*A*_1_)+*C*_5_+*H*_2_+*L*_2_>2*C*_3_+*U*
				*x*(*M*_4_+*M*_6_+*Q*_3_+*Q*_4_+3*H*_3_+*L*_3_+*M*_3_+*A*_2_+*B*_1_)+*Z*+*U*+*V*+*T*_1_+*T*_2_+*H*_4_+*L*_4_+*K*+*N*>2*C*_5_+2*C*_6_
*y* = y^**^, *z* = z^**^	0	0	Saddle point	Saddle point under any condition

## 4 Numerical simulation analysis

### 4.1 Initial assignment of model parameters

Heilongjiang Province is the largest agricultural-producing region in China. It accounts for one-ninth of the country's total grain output, indicating that one-ninth of the rice consumed by the Chinese population is produced in Heilongjiang. As such, it plays a crucial role as the “ballast” of food security. According to data from the Third Land Survey of Heilongjiang Province, the total arable land area in the region is 257.9 million mu, representing ~13% of the total arable land in China. Within Heilongjiang, there are 61,700 family farms, 95,000 farmers' cooperatives, and 114 farms and ranches managed by the Heilongjiang General Bureau of Reclamation. Taking Heilongjiang Province as a case study, and considering that the average operating area of family farms in the region is 271 mu, alongside the medium-scale operation of agricultural enterprises, this study examines the evolutionary trends in the behavioral decisions of multiple agents involved in agricultural green production under the dual-carbon objectives. The parameter data are primarily sourced from Chinese statistical yearbooks, major news reports, relevant literature, and data statements. Additionally, some data were obtained through expert consultation. The model parameters used in this study are expressed from the perspective of county-level governments, with the units in RMB per year. The specific values of the variable parameters are presented in Table 4.

**Table 4:** Variable parameter values.

**Parameters**	**Data source**	**Numerical value (million)**
*A*_1_	Drawing on the “Implementation Plan for Low - Carbon Loans in Ya'an City, Sichuan Province in 2022,” the People's Bank of China, through refinancing, supplies low-cost funds to corporate financial institutions. The aim is to guide these institutions to actively issue “low-carbon loans.” The Municipal Bureau of Economic and Information Technology and the Municipal Bureau of Finance, by means of the Industrial Development Fund and financial interaction funds, offer a 1 - percentage - point interest subsidy for “low-carbon loans” that conform to green development goals. After the financial subsidy, the actual interest rate borne by enterprises for these loans should not exceed 4.5%. For example, when the loan amount is estimated at 200,000 yuan, the subsidy amount is calculated as 200,000 × 1% = 2,000 yuan	0.2
*A*_2_	Drawing on the “Low-Carbon Loan Implementation Plan” issued by Ya'an City, Sichuan Province in 2022, the People's Bank of China provides low-cost funds to legal person financial institutions through re-lending, guiding them to actively issue “low-carbon loans.” The Municipal Bureau of Industry and Information Technology and the Municipal Finance Bureau provide a 1 percentage point interest subsidy for “low-carbon loans” that align with green development through industrial development funds and fiscal-financial interaction incentive funds. After fiscal interest subsidies, the actual loan interest rate borne by enterprises does not exceed 4.5%. 650,400 × 1% = 6,504	0.6504
*C*_1_	In 2022, Heilongjiang Province allocated 350,000 yuan to support the development of low-carbon agriculture, including the construction of low-carbon agricultural demonstration bases and monitoring and evaluation activities (40)	35
*C*_2_	Based on field surveys and an analysis of factors such as the enterprise's historical operational data, industry average levels, and anticipated operational efficiency, the estimated basic operating cost is ~200,000 yuan (41)	20
*C*_3_	According to the China Phosphatic Compound Fertilizer Industry Association, low-carbon raw materials (such as humic acid and microbial inoculants) command a premium of 20%−40% compared to traditional raw materials. Additionally, slow-release fertilizer production lines achieve a 15% reduction in energy consumption, although equipment depreciation costs increase by 8%−12%. Based on these factors, the estimated additional cost is ~100,000 yuan (42)	10
*C*_4_	Field research and through the village collective data statement learned that the basic operating cost per acre is about for 2400 yuan. The basic cost is 2,400 × 271 = 650,400 yuan	65.04
*C*_5_	Through market research, we know that the price of low-carbon agricultural materials is 10%−50% higher than non-low-carbon agricultural materials, taking the average value of 30%, taking wheat as an example, planting an acre of wheat, the application of non-low-carbon fertilizers and pesticides cost a total of 330 yuan	2.439
	The additional cost is about 271 × 30% × 300 = $24.39 million	
*M*_1_	The newly amended Article 60 of the “Pesticide Management Regulations” stipulates that for the irregular use of pesticides, which includes non-compliance with labeling requirements, the use of banned pesticides, the application of highly toxic pesticides for health pest control, on vegetables, melons, fruits, tea, mushrooms, or for aquatic plant pest control, the use of pesticides in protected areas of drinking water sources, the disposal of pesticides in protected areas of drinking water sources or rivers, as well as improper handling of pesticide packaging or cleaning of application equipment, enterprises and cooperatives shall be fined not < 50,000 yuan but not more than 100,000 yuan. For individuals, the fine shall be < 10,000 yuan. In this study, an average fine of 75,000 yuan is considered (43)	7.5
*M*_2_	According to the provisions of the “Clean Production Audit Measures” for relevant enterprises in Guangzhou City in 2022, enterprises that implement clean production for the first time and pass the audit will be awarded a subsidy of 150,000 yuan if they are rated as “Outstanding Clean Production Enterprises”; those rated as “Clean Production Enterprises” will receive a subsidy of 100,000 yuan; and those that pass the simplified clean production audit process will be granted a subsidy of 25,000 yuan. Based on this estimation, the total subsidy amounts to 90,000 yuan (44)	9
*M*_3_	In 2015, the use of low-toxicity pesticides in Jiaozhou City, Shandong Province, vegetables are subsidized 40 yuan per mu, the subsidy is 271 × 40 = 10.84 million yuan	1.084
*M*_4_	The newly amended Article 60 of the “Pesticide Management Regulations” stipulates that for the irregular use of pesticides, which includes non-compliance with labeling requirements, the use of banned pesticides, the application of highly toxic pesticides for health pest control, on vegetables, melons, fruits, tea, mushrooms, or for aquatic plant pest control, the use of pesticides in protected areas of drinking water sources, the disposal of pesticides in protected areas of drinking water sources or rivers, as well as improper handling of pesticide packaging or cleaning of application equipment, enterprises and cooperatives shall be fined not < 50,000 yuan but not more than 100,000 yuan. For individuals, the fine shall be < 10,000 yuan. In this study, an average fine of 75,000 yuan is considered (45)	7.5
*M*_5_	In 2022, Heilongjiang Province will implement a green and low-carbon manufacturing initiative, carrying out energy-saving and carbon-reduction green upgrades for industrial enterprises with annual energy consumption of 5,000 tons of standard coal or more. Enterprises that achieve annual energy savings of 1,000 tons of standard coal or more, reduce carbon emissions by 2,500 tons or more, or achieve energy consumption per unit of product at the national benchmark level will be awarded a grant of 1 million yuan. Based on this estimate, the subsidy amounts to 30,000 yuan	3
*M*_6_	In 2022, Heilongjiang Province will support the development of agricultural and sideline products and “time-honored brands” and other distinctive Heilongjiang products by “increasing variety, improving quality, and creating brands.” Product packaging will be designed according to market demand to significantly enhance the visibility and reputation of Heilongjiang products. Enterprises that purchase creative design services may apply for a one-time subsidy, with the subsidy amounting to 50% of the actual amount incurred under the service contract, up to a maximum of 200,000 yuan. Based on this estimate, the subsidy would be 100,000 yuan	10
*N*	The green production of agricultural products from the survey is 2-4 yuan per kilogram more expensive than ordinary agricultural products, and the average yield of three plots of soybeans of Longken 3092 in 2021 in the construction farm of Beidahuang Group is about 300 kg per mu. The extra income is 300 × 3 × 271 = 243,900 yuan	24.39
*P*	According to reference (37), in 2020, the central government will arrange 1.6 billion yuan of funds to support the implementation of conservation farming in three northeastern provinces and regions with an area of 40 million mu, and the cost of environmental management will be 16,000,000,000/4,000,000 × 271 = 108,400 yuan	10.84
*Q_3_*	Drawing on Finland's carbon - tax policy with a tax rate of $30 per ton of carbon dioxide, and based on relevant literature, the carbon emissions of Jilin Province's plantation industry in 2018 were estimated. The total carbon emissions were ~3.46 million tons, with a cultivated-land area of 104.85 million mu. Thus, the carbon emission per mu was calculated as 346/10,485 = 0.032 tons. Subsequently, considering the relevant factors, the carbon tax was computed as 30 × 6.87 × 0.032 × 271 = 1,787 yuan (46)	0.1787
*U*	According to the Notice of the Ministry of Finance and the State Administration of Taxation on the VAT Exemption Policy for Agricultural Production Materials, certain agricultural materials are exempt from VAT, enabling agricultural material companies to offer discounts to farms. Based on field research and interviews with farm personnel, the discount is estimated to be 20,000 yuan	0.3793

### 4.2 Evaluation of the implementation effect of the initial policy

**Figure 3:**
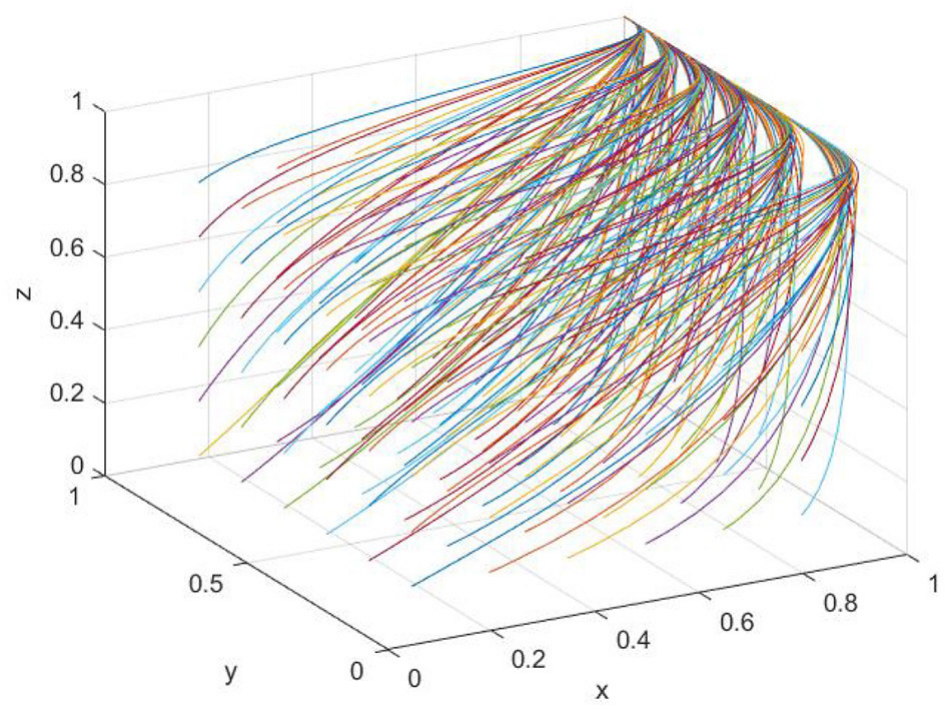
Convergence toward full compliance equilibrium.

After analyzing the evolutionary stability between the government and agribusinesses, as well as between agribusinesses and farms, the stability points and their corresponding conditions were identified. To explore the influence of parameter variations on the evolutionary trajectories of the three parties, these parameters were integrated into a unified game system. To assess the evolution and stability points under different initial conditions, multiple evolutionary simulations were conducted with varying initial probabilities to observe the system's developmental trajectories. The initial decision-making probability increment was set at 0.15, and a total of 216 simulations were performed. As shown in Figure 3, under the current policy framework, the system ultimately converges to the (1, 1, 1) equilibrium point. This outcome indicates that the government adopts regulatory measures, agricultural enterprises choose to produce low-carbon agricultural inputs, and farms opt to utilize such low-carbon materials. This convergence suggests that the current policy framework effectively promotes the coordinated adoption of low-carbon strategies among all three stakeholders, which is vital for achieving the dual-carbon objectives in the agricultural sector. The stability at this point implies a self-sustaining equilibrium, wherein the incentives and constraints embedded within the game system encourage each party to act in ways that support sustainable agricultural development.

This result can be attributed to the broader context of China's dual-carbon goals. Under such circumstances, proactive regulatory interventions by the Chinese government can lead to more favorable outcomes, with the overall benefits outweighing those of non-regulatory approaches. Through government regulation, agricultural enterprises and farms are more likely to decide on producing low-carbon inputs and utilizing low-carbon agricultural materials, thereby responding effectively to national dual-carbon initiatives while also gaining tangible benefits. Consequently, the (1, 1, 1) equilibrium aligns with 'China's long-term development goals. This indicates that the current policy framework effectively fosters the joint pursuit of low-carbon strategies among all stakeholders, playing a pivotal role in achieving the dual-carbon objectives within the agricultural domain. The stability at this point represents a self-perpetuating equilibrium, where the incentives and constraints within the system mutually reinforce sustainable development in agriculture.

### 4.3 Analysis of the effect of initial values of *x, y, z* on the evolution of the strategy

**Figure 4:**
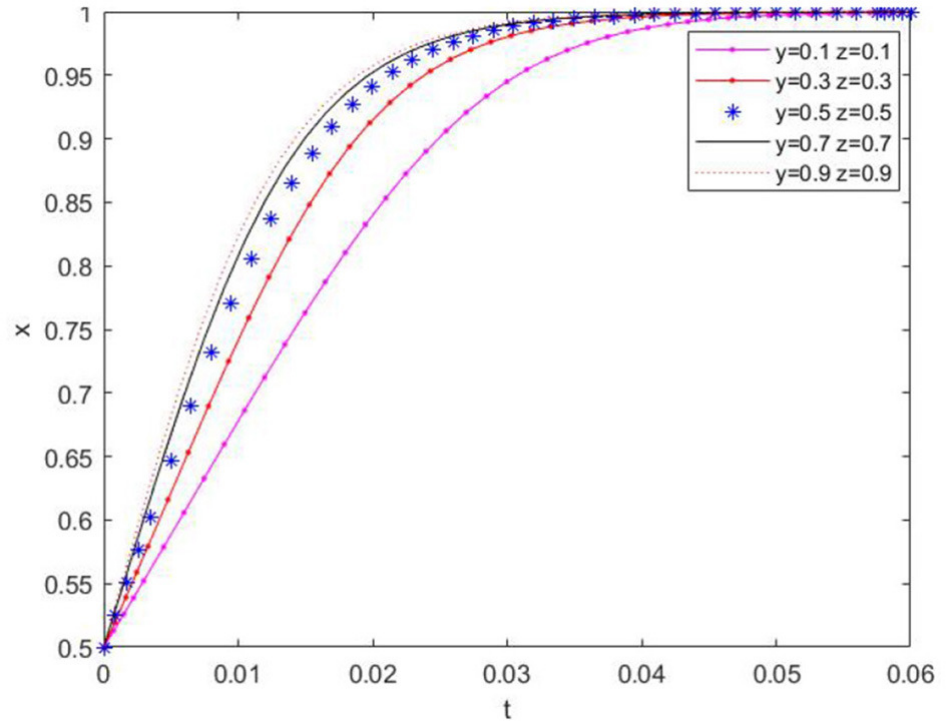
Effect of yz initial value change on x-value evolution path.

**Figure 5:**
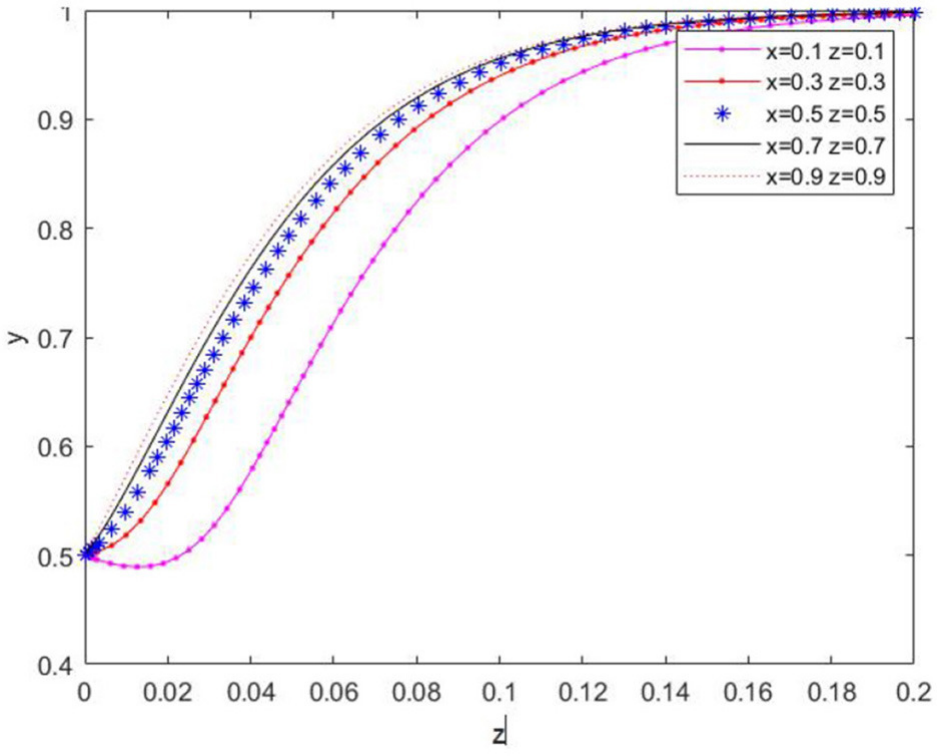
Effect of xz initial value change on y-value evolution path.

**Figure 6:**
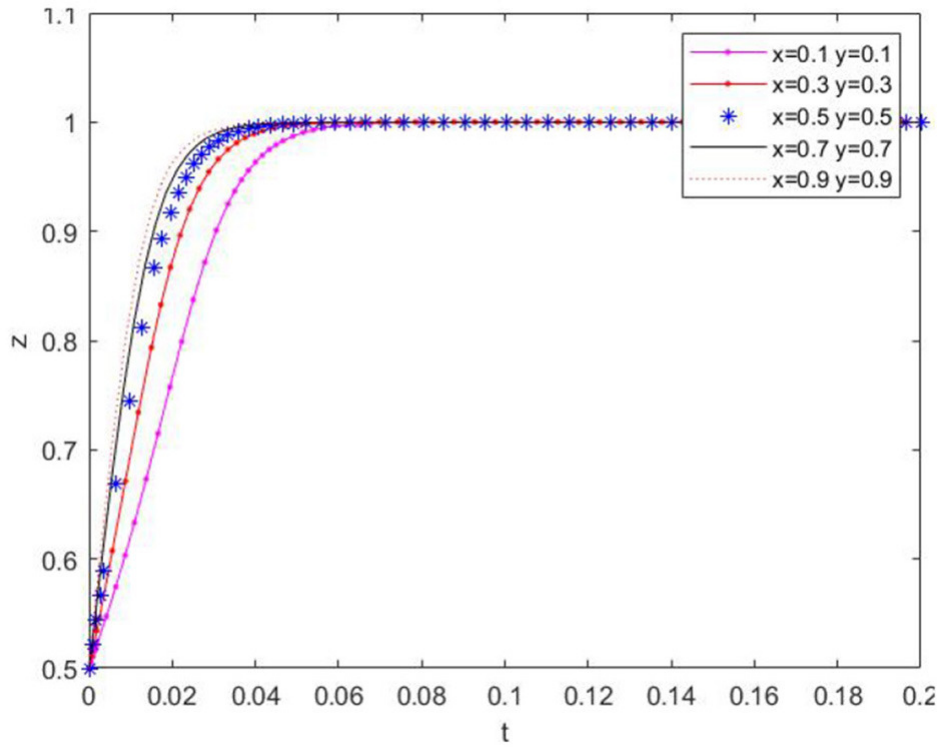
Effect of the change of xy initial value on the evolutionary path of z value.

**Figure 7:**
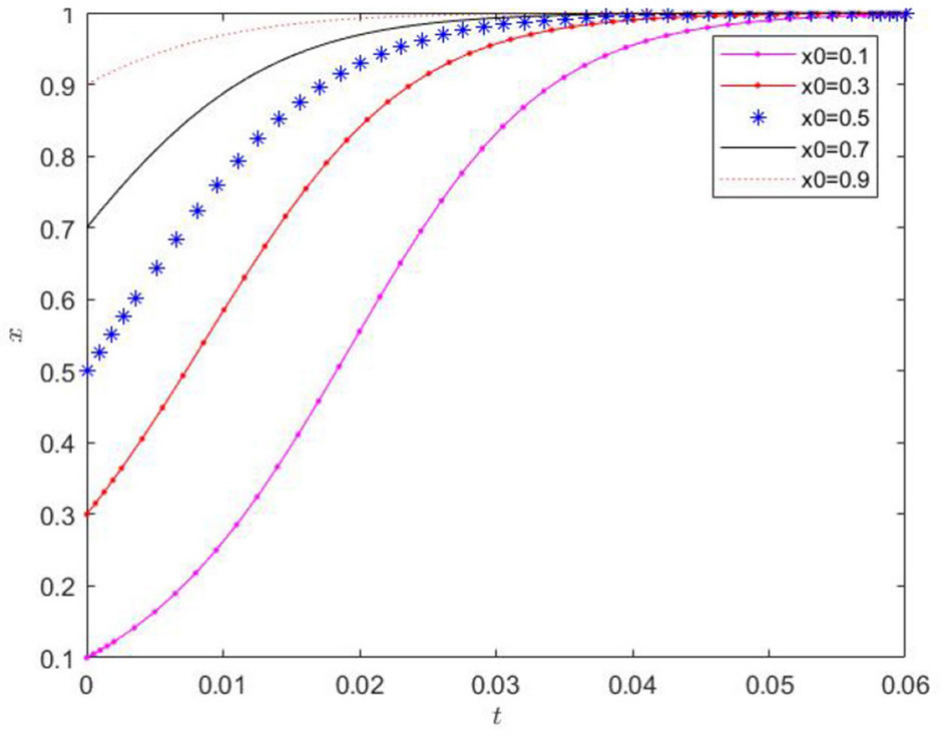
Effect of the change of x initial value on its evolution.

**Figure 8:**
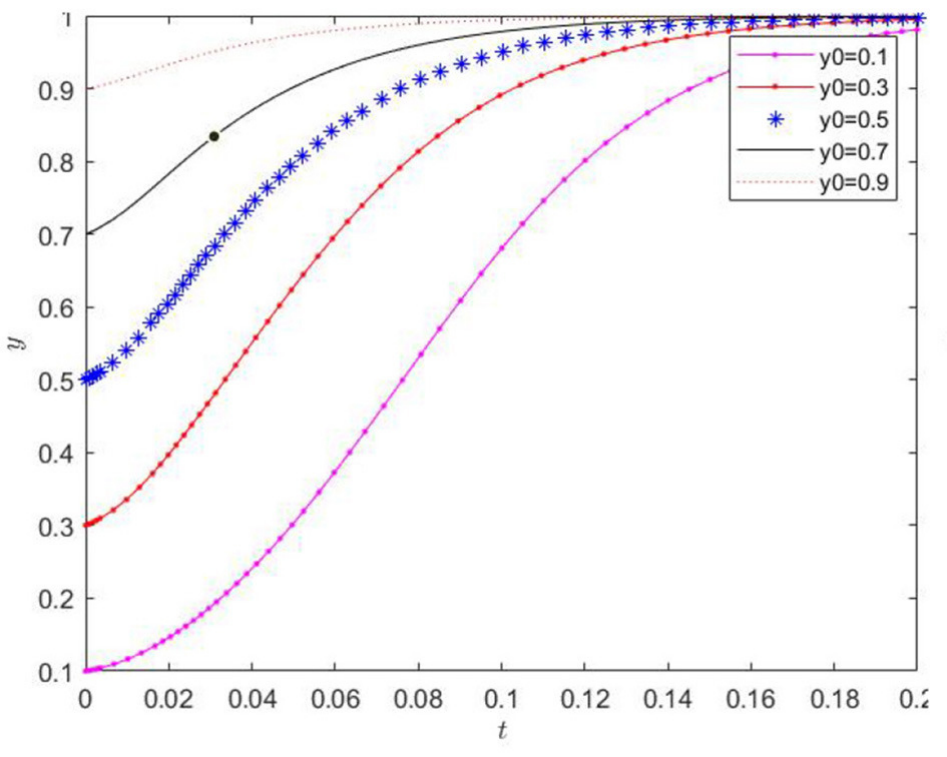
Effect of the change of initial value of y on its evolution.

**Figure 9:**
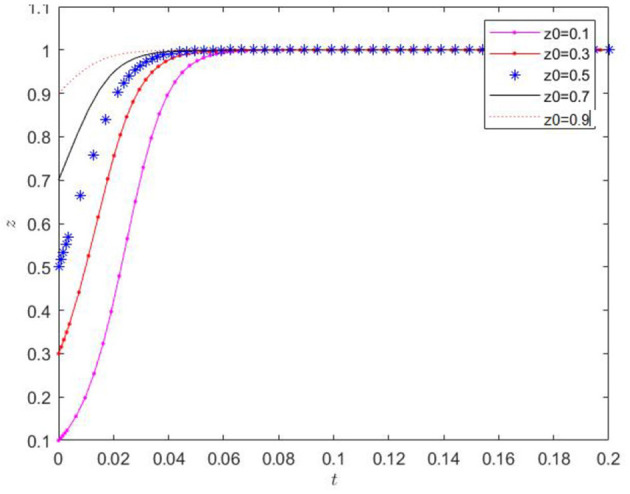
Effect of the change of initial value of z on its evolution.

(1) Based on the aforementioned conditions, the initial value of *x* was set to 0.5, and the initial values of *y* and *z* were varied to examine their impact on the temporal change of *x*, as presented in Figure 4. From the figure, it can be observed that the convergence rate of *x* is influenced by the initial values of *y* and *z*. However, ultimately *x* converges to 1, signifying that the government will eventually adopt the “regulatory” strategy.

(2) Similarly, the initial value of *y* was set to 0.5, and the initial values of *x* and *z* were altered to verify the temporal evolution of *y*, as depicted in Figure 5. Evidently, the initial values of *x* and *z* affect the convergence rate of *y*, yet *y* ultimately converges to 1. This indicates that agricultural enterprises are influenced by the choices of the other two parties but will eventually evolve to the strategy of “producing low-carbon agricultural materials.”

(3) With the initial value of *z* set at 0.5, the initial values of *x* and *y* were selected to assess their effect on the temporal change of *z*, as shown in Figure 6. It can be seen that the initial values of *x* and *y* influence the convergence rate of *z*, and *z* ultimately converges to 1. This implies that farms will eventually choose the strategy of “applying low-carbon agricultural materials” during the evolutionary process.

(4) When the initial values of y and *z* were fixed, and the initial value of *x* was randomly chosen with 0.1 as the lower limit, the influence of the initial value of x on the evolutionary trend of *x* was verified, and the results are presented in Figure 7. The ratio of the government's regulatory decisions consistently exhibits a monotonically increasing trend over time. The initial value only has a minor impact on the rate of change of the *x*-value. After a certain period of evolution, the *x*-value approaches the extreme value of 1. The initial conditions can lead to the government eventually making regulatory decisions uniformly.

(5) When the initial values of *x* and *z* were fixed, and the initial value of *y* was randomly selected with 0.1 as the lower limit, the influence of the initial value of *y* on the evolutionary trend of *y* was verified, and the results are shown in Figure 8. The proportion of agricultural enterprises adopting the production of low-carbon agricultural materials always shows a monotonically increasing trend with time. The initial value only marginally affects the rate of change of the *y*-value. After a certain time of evolution, the *y*-value approaches the extreme value of 1. The initial conditions can cause all agricultural enterprises to eventually adopt the production of low-carbon agricultural materials.

(6) When the initial values of *x* and *y* were fixed, and the initial value of *z* was randomly chosen with 0.1 as the lower limit, the effect of the initial value of *z* on the evolutionary trend of *z* was verified, and the results are presented in Figure 9. The proportion of farms applying low-carbon agricultural decisions consistently shows a monotonically increasing trend over time. The initial value only slightly affects the rate of change of the *z*-value. After a certain period of evolution, the *z*-value approaches 1. The initial conditions can enable all farms to eventually implement low-carbon farming decisions.

### 4.4 Analysis of the influence of parameters on strategy evolution

(1) The influence of variations in government subsidies for the application of low-carbon agricultural materials on the evolutionary process

**Figure 10:**
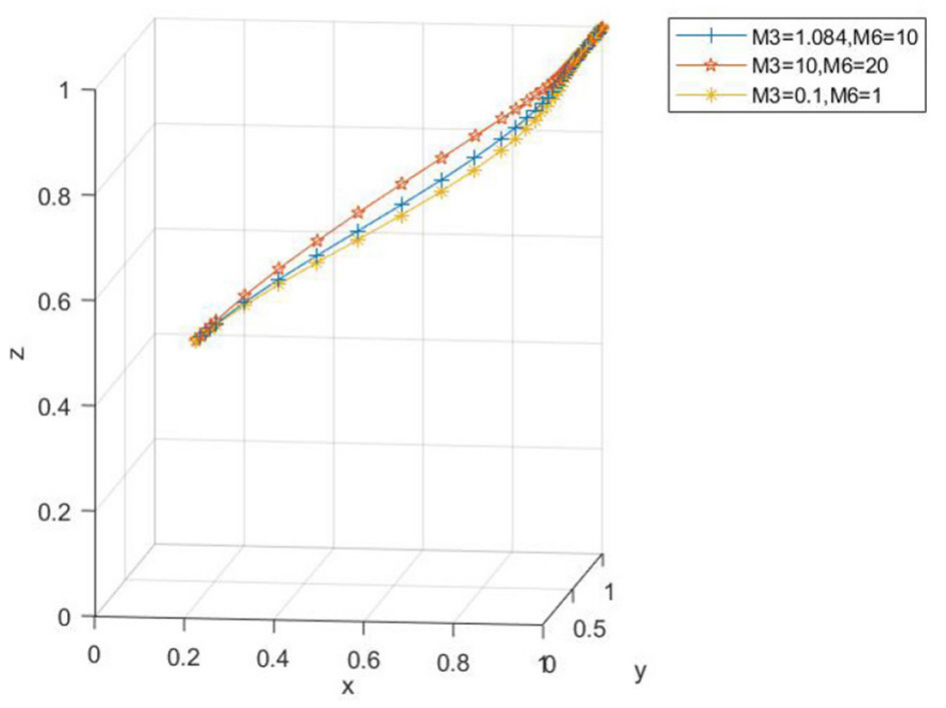
Spatial map of the impact of farm subsidy changes on evolutionary paths.

**Figure 11:**
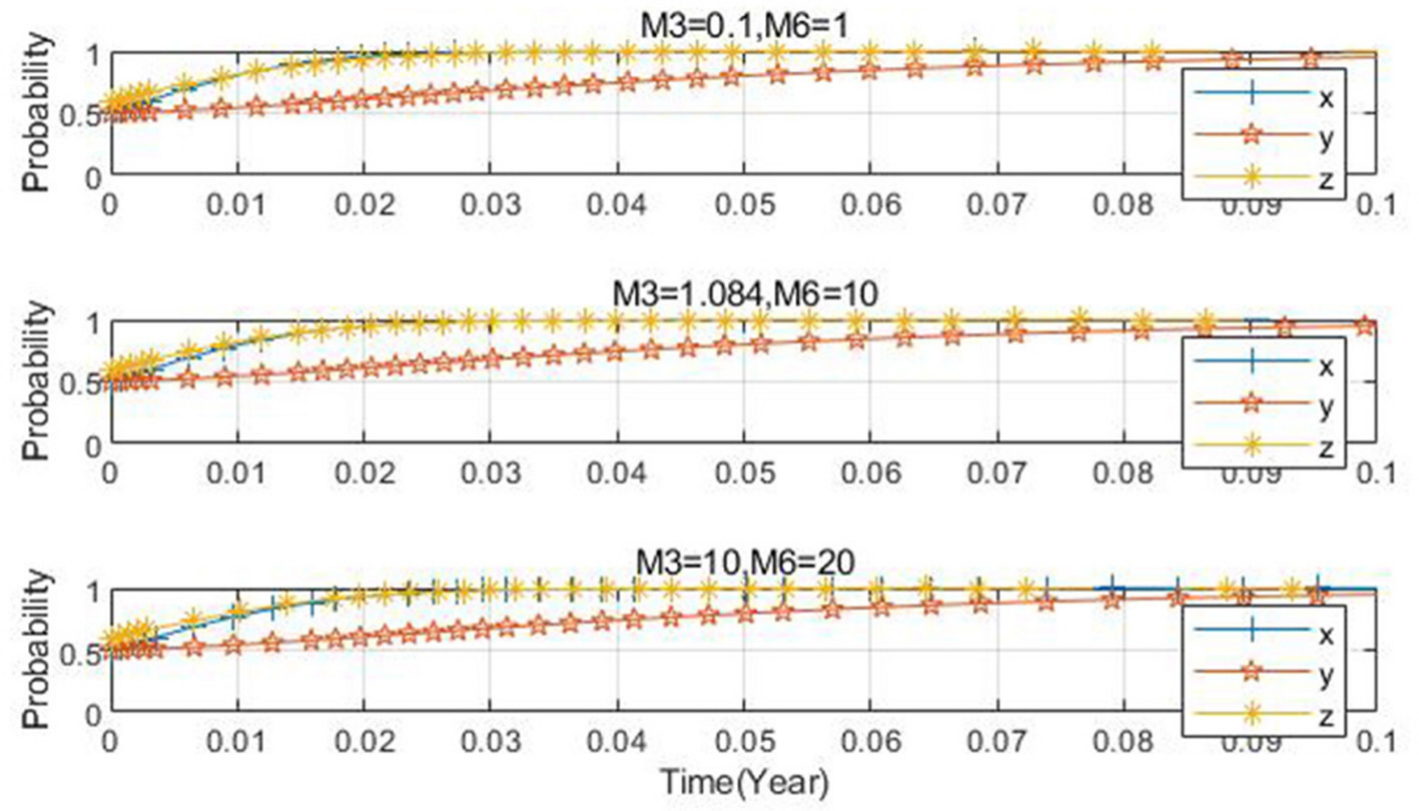
Impact of farm subsidy changes on each subject.

Taking *x*_0_ = 0.2, *y*_0_ = 0.2, *z*_0_ = 0.5 as an illustration, the parameters are set as *M*_3_ = 1.084, *M*_6_ = 10; *M*_3_ = 10, *M*_6_ = 20; *M*_3_ = 0.1, *M*_6_ = 1, respectively. As is evident from Figure 10, when the subsidy is either relatively large or small, although the three-party evolution ultimately leads to the ideal state, there exists a disparity in the convergence speed. Specifically, when the subsidy is too small, a longer time is required for convergence to the ideal state. As the subsidy amount increases, the convergence speed of the three-party evolutionary system accelerates, and the time needed to reach the ideal state diminishes, eventually evolving to the desired state. Figure 11 reveals that with the increase in subsidies for farms applying low-carbon farming materials, the probability of the government choosing to regulate decelerates slightly, while the probability of farms applying low-carbon farming materials accelerates slightly. This indicates that as the subsidy rises, it imposes a significant financial burden on the government and provides a stronger incentive for farms to apply low-carbon farming materials.

(2) The impact of changes in farm carbon-tax rebates on the evolutionary process

**Figure 12:**
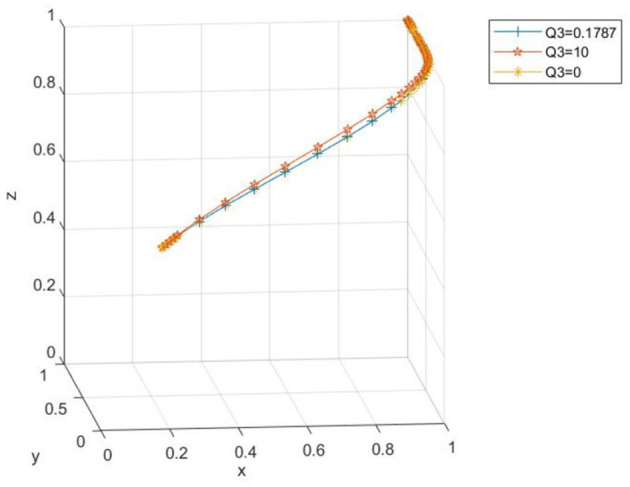
Spatial diagram of the impact of carbon tax rebate changes on the evolutionary path.

**Figure 13:**
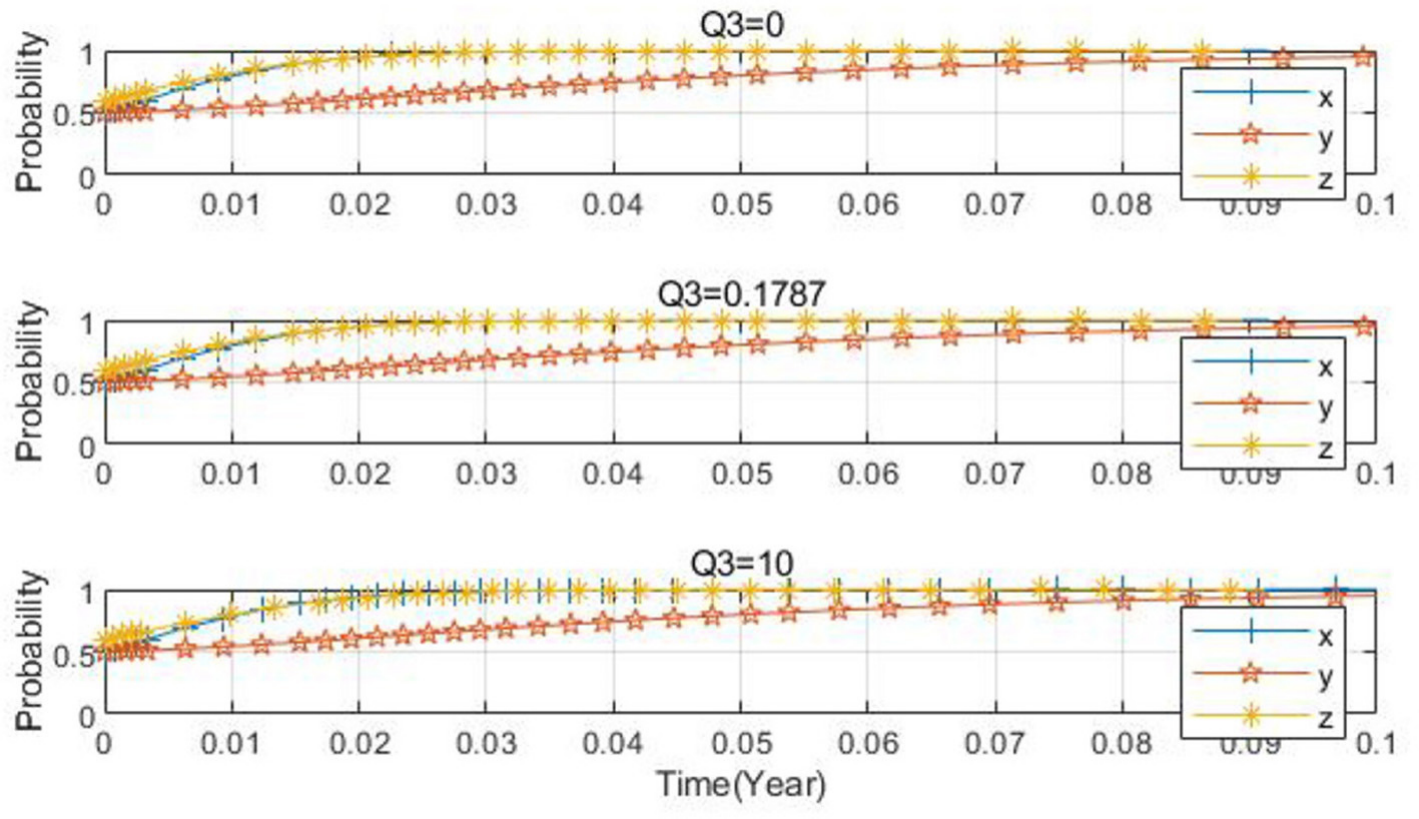
Impact of carbon tax rebate changes on each subject.

Taking *x*_0_ = 0.2, *y*_0_ = 0.2, *z*_0_ = 0.5 as examples, the parameters are set as *Q*_3_ = 0.1787, *Q*_3_ = 10, and *Q*_3_ = 0, respectively. From Figure 12, it can be observed that when the carbon-tax rebate is either relatively large or small, although the three-party evolution culminates in the ideal state, there is a difference in the convergence speed. When the carbon-tax rebate is too small, a more extended period is needed to converge to the ideal state. As the carbon-tax rebate increases, the convergence speed of the tripartite evolutionary system quickens, and the time required to reach the ideal state decreases, with the system ultimately evolving to the ideal state. Figure 13 shows that as the carbon-tax rebate increases, the probability that the government chooses to regulate decelerates slightly, and the probability that farms apply low-carbon farming materials accelerates slightly. This implies that as the carbon-tax rebate for farms grows, it places a substantial financial burden on the government and offers a greater incentive to farms.

(3) The impact of different penalty intensities on the government, agribusinesses, and farms

**Figure 14:**
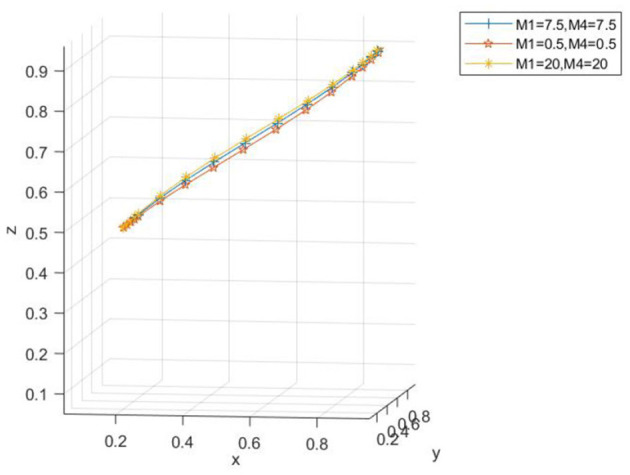
Spatial diagram of the impact of the change in fines on the evolutionary path.

**Figure 15:**
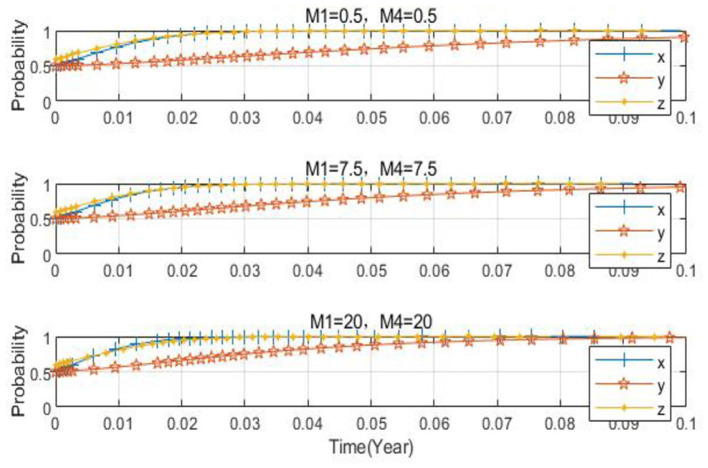
Impact of the change in fines on the subjects.

Taking *x*_0_ = 0.2, *y*_0_ = 0.2, *z*_0_ = 0.5 as examples, the parameters are set as *M*_1_ = 7.5, *M*_4_ = 7.5; *M*_1_ = 20, *M*_4_ = 20; *M*_1_ = 0.5, *M*_4_ = 0.5, respectively. As can be seen from Figure 14, when the penalty is either relatively large or small, although the three-party evolution results in the ideal state, there is a variance in the convergence speed. When the penalty is too small, a longer time is needed to converge to the ideal state. As the fine amount increases, the convergence speed of the three-party evolutionary system speeds up, and the time required to reach the ideal state reduces, eventually evolving to the ideal state. Figure 15 demonstrates that as the government fines for enterprises producing non-low-carbon agricultural materials and farms applying non-low-carbon agricultural materials increase, the probability of agribusinesses adopting low-carbon agricultural materials and the probability of farms adopting low-carbon agricultural materials also increase.

(4) The influence of the low-carbon agricultural market on agribusinesses and farms

**Figure 16:**
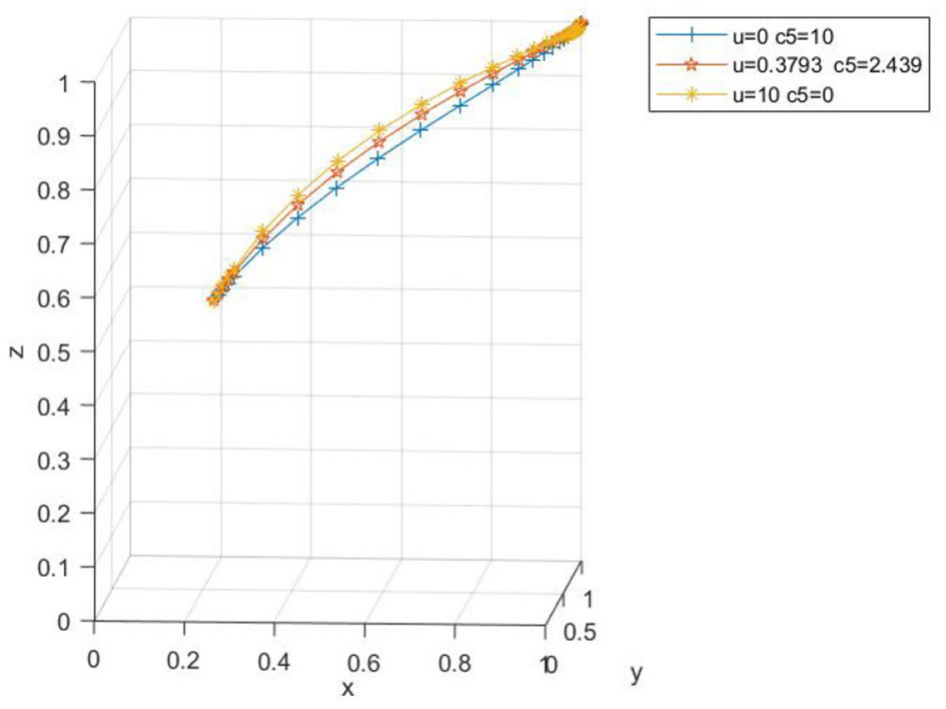
Spatial diagram of the impact of low carbon agricultural market on evolutionary path.

**Figure 17:**
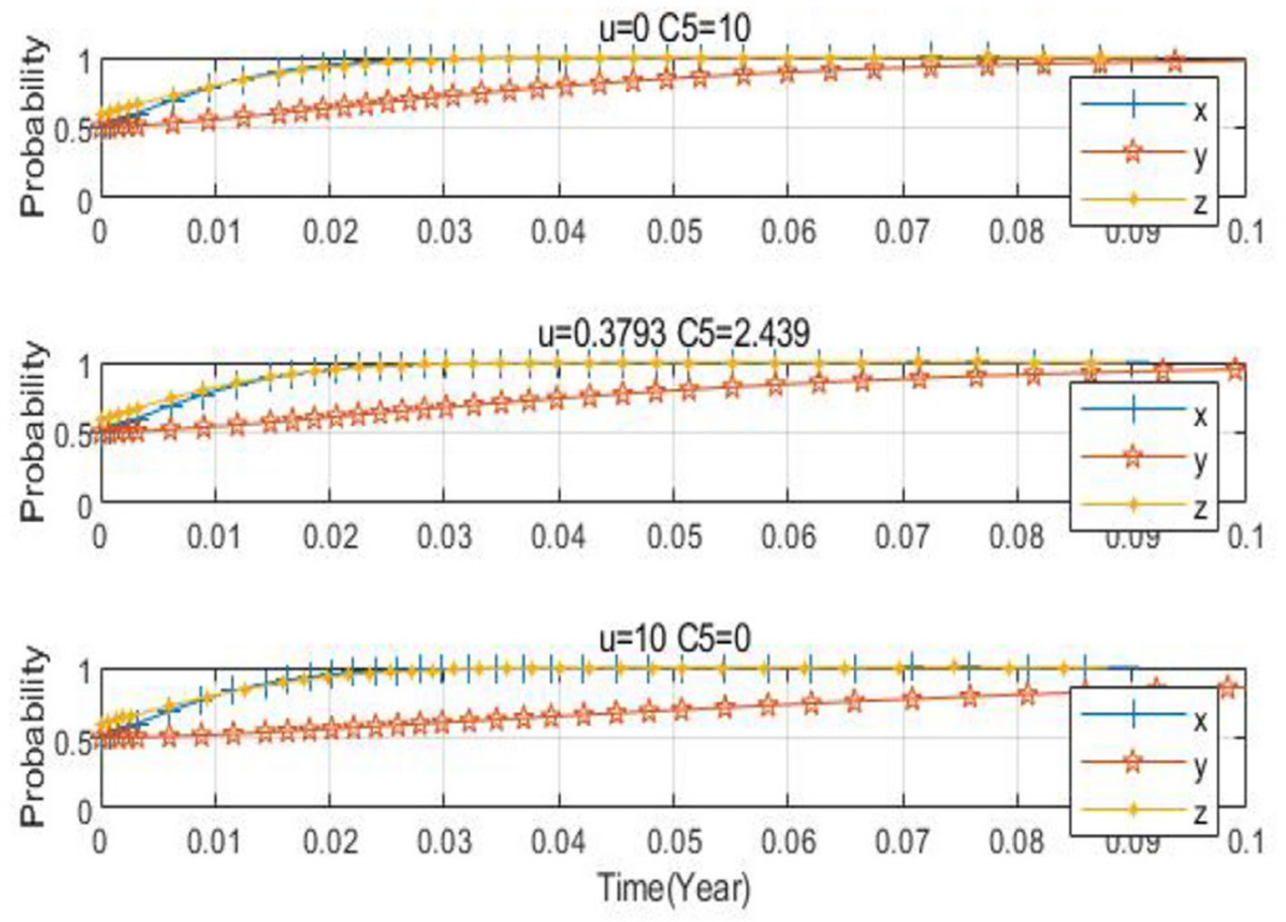
Impact of low carbon agricultural market on each subject.

When the probability of agricultural enterprises choosing to produce low-carbon agricultural materials is high, due to the increase in the number of enterprises producing low-carbon agricultural materials, the price concessions offered to farms will be lower, and the extra cost borne by farms will be higher. Taking *x*_0_ = 0.2, *y*_0_ = 0.8, *z*_0_ = 0.5 as an example, the parameters are set as *U* = 0.3793, *C*_5_ = 2.439; *U* = 10, *C*_5_ = 0; *U* = 0, *C*_5_ = 10, respectively. From Figures 16, 17, it can be discerned that when the concession increases within the range of 0–100,000 yuan and the extra cost decreases within the range of 100,000 yuan−0, the three-party evolution result is ideal, and the convergence rate gradually rises. This indicates that as the price concession increases and the extra cost for farms decreases, the probability of farms applying low-carbon agricultural materials is on the increase.

## 5 Discussion and conclusion

### 5.1 Conclusion

The data presented in this paper originates from the Heilongjiang region of China, this paper constructs a three-party evolutionary game model involving the government, village collectives, and farmers using evolutionary game theory. It conducts replicated-dynamic equation analysis, evolutionary stability analysis, and numerical simulation experiments, leading to the following main conclusions:

(1) From the decision-replication dynamic equations, it can be deduced that the proportion of the government's regulatory decisions is related to the proportion of agricultural enterprises' decisions to produce low-carbon agricultural resources and the proportion of farms' decisions to apply low-carbon agricultural resources. The proportion of agricultural enterprises' decisions to produce low-carbon agricultural resources is associated with the proportion of the government's regulatory decisions and the proportion of farms' decisions to apply low-carbon agricultural resources. The proportion of farms' decisions to apply low-carbon agricultural resources is linked to the proportion of the government's regulatory decisions and the proportion of agricultural enterprises' decisions to produce low-carbon agricultural resources. In essence, the decision of any one party is influenced by the decisions of the other two parties.

(2) Based on the numerical simulation results, it can be concluded that the evolution process toward the ideal state in the three-party system exhibits the following characteristics:

The government adopts regulation, agricultural input enterprises implement low-carbon agricultural input production modes, and farms apply low-carbon inputs. Variations in the initial values of the government's regulatory proportion, the proportion of agricultural input enterprises producing low-carbon inputs, and the proportion of farms applying low-carbon inputs influence only the convergence speed but do not affect the final evolutionary outcome, which ultimately converges to the ideal state. When government subsidies for farms using low-carbon inputs increase within the range of 50,000–1,000,000 yuan, the convergence speed of the system accelerates, reducing the time required to reach the ideal state. However, subsidies exceeding 1,000,000 yuan impose a financial burden on the government, thereby prolonging the evolution toward the ideal state. Similarly, when the carbon tax rebates for farms range from 3,000 to 90,000 yuan and those for agricultural input enterprises range from 1,000 to 200,000 yuan, the convergence speed of the system is enhanced, and the time to achieve the ideal state is shortened. Conversely, if the rebates exceed 90,000 or 200,000 yuan, respectively, the time for the government to evolve toward the ideal state increases.

Furthermore, increasing penalties for agricultural input enterprises not adhering to low-carbon production modes within the range of 2,000–300,000 yuan, and penalties for farms not applying low-carbon inputs within the range of 50,000–100,000 yuan, both lead to faster convergence of the three-party system toward the ideal state, reducing the required adaptation time. Under conditions where subsidies range from 2,000 to 10,000 yuan and additional costs decrease from 250,000 to 150,000 yuan, the system also converges to the ideal state, with the convergence speed gradually increasing. This indicates that higher subsidies and reduced additional costs increase the likelihood of farms adopting low-carbon inputs. In summary, while this study uses Heilongjiang Province as a case example, it is essential to explicitly acknowledge this regional limitation within the discussion section to clarify the scope of generalization. I encourage you to revise the manuscript carefully following these insights.

### 5.2 Discussion and recommendations

This study reveals the evolutionary pathways and regularities of decision-making behaviors among the government, agricultural input enterprises, and farms under the context of carbon neutrality. It also identifies the equilibrium and stability conditions under which these subjects' decisions reach the ideal state, supported by numerical simulations. These findings offer valuable theoretical insights and practical guidance for government regulatory policymaking, the transformation of agricultural input enterprises toward low-carbon production modes, and the transition of farms to applying low-carbon inputs. Based on the game-theoretic outcomes, the following three recommendations are provided:

From the government's perspective, the government serves as a strong promoter of both enterprises adopting low-carbon agricultural input production modes and farms implementing low-carbon inputs. Promoting ecological farms can safeguard national food security and the effective supply of key agricultural products while alleviating ecological environmental pressure. Regulatory authorities can increase subsidies to farms within the range of 50,000–500,000 yuan and enhance carbon tax rebates within 3,000–50,000 yuan. Additionally, penalties for agricultural input enterprises not adopting low-carbon production modes can be increased within the range of 2,000–300,000 yuan, incentivizing enterprises to shift toward low-carbon production. Similarly, penalties for farms not applying low-carbon inputs can be raised within 50,000–100,000 yuan. Moreover, as some farms and enterprises lack sufficient awareness of carbon neutrality and are primarily motivated by short-term interests, the government should improve mechanisms for promoting knowledge of carbon neutrality.

From the perspective of agricultural input enterprises, these entities should actively respond to government initiatives as intermediaries in the tri-party game. By offering preferential prices for low-carbon inputs and increasing their subsidies within the range of 2,000–100,000 yuan, enterprises can facilitate the transition from non-low-carbon to low-carbon production modes. In the context of global carbon neutrality efforts, enterprises should not prioritize immediate profits at the expense of sustainable practices. As government regulation intensifies and farms increasingly favor low-carbon inputs, enterprises producing low-carbon inputs will hold a competitive advantage.

From the farms' perspective, establishing long-term stable strategic partnerships with low-carbon input enterprises is essential. Such collaborations can help farms secure higher-quality, more affordable, and abundant supplies of agricultural inputs. Since farm behavior directly impacts the achievement of agricultural carbon neutrality, farms should actively respond to national calls, enhance their understanding of carbon neutrality-related knowledge, and transition from applying non-low-carbon inputs to low-carbon inputs. Not only can applying low-carbon inputs bring subsidies and other benefits, but it also contributes to China's early realization of carbon neutrality. Additionally, using low-carbon inputs can improve land quality, thereby generating better economic returns.

In summary, this study primarily relies on empirical data from Heilongjiang Province. However, due to limitations in the scope of the survey area and data availability, there are certain shortcomings in the research. For example, the study is mainly based on data from a specific region within Heilongjiang Province. While this aligns with the characteristics of China's main grain-producing black soil region, regional specificities such as land protection policies and large-scale management practices may restrict the universality of the proposed driving strategies. Future research should consider broadening the data sources to enhance the general applicability of the findings and better support farmers in their efforts to achieve carbon emission reductions.

## Data Availability

The original contributions presented in the study are included in the article/supplementary material, further inquiries can be directed to the corresponding authors.
